# adhesiomeR: a tool for *Escherichia coli* adhesin classification and analysis

**DOI:** 10.1186/s12864-024-10525-6

**Published:** 2024-06-17

**Authors:** Katarzyna Sidorczuk, Michał Burdukiewicz, Klara Cerk, Joachim Fritscher, Robert A. Kingsley, Peter Schierack, Falk Hildebrand, Rafał Kolenda

**Affiliations:** 1 Quadram Institute Bioscience, Norwich Research Park, Norwich, UK; 2 Earlham Institute, Norwich Research Park, Norwich, UK; 3 Department of Bioinformatics and Genomics, Faculty of Biotechnology, University of Wrocław, Wrocław, Poland; 4 Institute for Biotechnology, Brandenburg University of Technology (BTU) Cottbus-Senftenberg, Senftenberg, Germany; 5 Clinical Research Centre, Medical University of Białystok, Białystok, Poland; 6 Institute of Biotechnology and Biomedicine, Autonomous University of Barcelona, Cerdanyola del Vallès, Spain; 7 Depertment of Biological Sciences, University of East Anglia, Norwich, UK; 8 Department of Biochemistry and Molecular Biology, Faculty of Veterinary Medicine, Wrocław University of Environmental and Life Sciences, Wrocław, Poland

**Keywords:** *Escherichia coli*, Adhesins, Adhesiome, Fimbriae, Adhesion, Pathotype, Virulence factor

## Abstract

Adhesins are crucial factors in the virulence of bacterial pathogens such as *Escherichia coli*. However, to date no resources have been dedicated to the detailed analysis of *E. coli* adhesins. Here, we provide adhesiomeR software that enables characterization of the complete adhesin repertoire, termed the adhesiome. AdhesiomeR incorporates the most comprehensive database of *E. coli* adhesins and facilitates an extensive analysis of adhesiome. We demonstrate that adhesiomeR achieves 98% accuracy when compared with experimental analyses. Based on analysis of 15,000 *E. coli* genomes, we define novel adhesiome profiles and clusters, providing a nomenclature for a unified comparison of *E. coli* adhesiomes.

**Supplementary Information:**

The online version contains supplementary material available at 10.1186/s12864-024-10525-6.

## Background

*Escherichia coli* is a bacterial species present as a common gut commensal or pathogen in humans and other animals and can survive in the environment during transmission between hosts [1, 2]. Distinct genotypes cause a wide range of intestinal and extraintestinal diseases, e.g., inflammatory bowel disease (IBD), with millions of infections annually [3]. Moreover, antimicrobial-resistant (AMR) *E. coli* strains have emerged due to overuse of antibiotics and are now a leading cause of death, necessitating novel treatments of *E. coli* infections [4, 5]. *E. coli* strains are classified into distinct pathotypes based on the presence of certain virulence-associated factors (VAFs). For example, enterotoxigenic *E. coli* (ETEC) produces specific enterotoxins and colonization factors (CFs) causing diarrhoea, whereas uropathogenic *E. coli* (UPEC), which may induce urinary tract infections, possesses various fimbriae and secreted VAFs [6, 7]. ETEC and enteropathogenic *E. coli* (EPEC) infections in low- and middle-income countries (LMICs) cause disease outbreaks requiring interventions. ETECs are estimated to cause about 220 million cases of diarrhoea, 75 million affecting children under 5 years of age [8]. The high level of mortality, especially in children, makes the development of the ETEC vaccine the WHO’s primary strategic goal [8].

One of the promising targets for novel intervention strategies are adhesins, structures that mediate bacterial attachment to various surfaces, including host cells [9, 10]. They are a key factor enabling host colonization and pathogenesis of bacteria and therefore, ideal candidates for the development of new treatments. Adhesins have been used successfully as vaccines, for example against recurrent urinary tract infections, which confirmed their therapeutic potential [11]. Another promising strategy encompasses anti-adhesion treatments, which focus on the development of factors that specifically block adhesion. These approaches require a detailed knowledge of adhesin properties and their distribution in commensal and pathogenic strains [12].

Proteinaceous adhesins can be classified into fimbrial and nonfimbrial [13]. Fimbriae are supramolecular hair-like protein structures usually encoded by an operon consisting of multiple co-regulated genes. Many fimbriae depend upon usher and chaperone proteins for biogenesis of structural subunits and tip adhesins whose expression is controlled by common regulatory elements [14]. The nonfimbrial adhesins are mainly represented by outer membrane proteins such as those of the autotransporter family [14].

Since adhesins are regarded as important virulence factors of *E. coli*, they are often included in tools dedicated to the analysis of virulence factors, such as VFDB [15], Victors [16], and Virulence Finder [17]. However, the role of adhesins as VAFs has been studied for only fraction of them and as a result, many adhesin sequences are not present in available databases. In our previous studies of hemolytic *E. coli* we found that the repertoire of specific adhesin genes co-occurred in *E. coli* strains harbouring either alpha- or entero-hemolysin [18]. Furthermore, specific repertoires were associated with alpha-hemolytic *E. coli* isolated from different hosts (humans or farm animals) [19]. To enable further analysis of adhesiomes, we expanded our collection of adhesin sequences into the most comprehensive, manually curated set of *E. coli* adhesins.

Here, we present adhesiomeR, an open-source tool available as an R package and a web server. It includes a collection of 525 *E. coli* adhesin genes grouped into 102 systems and allows qualitative inspection of genome-encoded adhesin repertoires in (meta)genomic data. Results are available on both gene and adhesin system level, the latter encoded across multiple genes within a gene cluster. Based on the analysis of 15,000 *E. coli* genomes, we propose a novel adhesiome profiling nomenclature that enables reproducible comparison of *E. coli* adhesiome types between studies. AdhesiomeR classifies analysed *E. coli* strains into these novel adhesiome profiles and clusters, providing valuable insights into their adhesion and pathogenic potential. To our knowledge, it is the first approach to facilitate analysis of the complete repertoire of *E. coli* adhesins, the adhesiome.

## Results

### Overview of the adhesiomeR tool

The search for adhesin genes in adhesiomeR is implemented using BLAST + (blastn algorithm) [20] due to its availability and ease of usage. The genes are divided into three groups depending on their identity to each other: (i) highly similar—genes with > 95% nucleotide (nt) identity to representative adhesins in our database, (ii) moderately similar—genes with 75–95% nt identity, and (iii) unrelated—genes that exhibited remote similarity or no similarity to other adhesins in our database (identity < 75%) (Fig. S1). AdhesiomeR does not distinguish between genes at an identity level higher than 95% and reports highly similar genes as groups to minimize the effects of incomplete sequences or sequencing errors on the results. If a moderately similar gene matches multiple adhesin genes, adhesiomeR selects the one with the highest percent identity. By comparing localization within the genome (Fig. S2), adhesiomeR identifies multiple copies of the same gene.

**Fig. 1:**
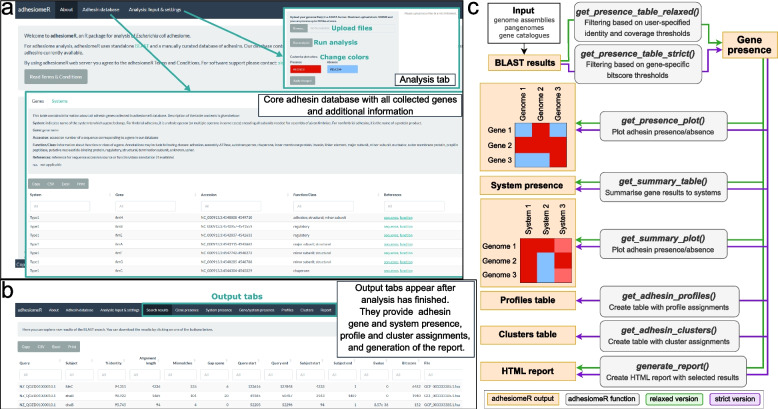
AdhesiomeR web server and R package. **a** Web server home page presents short tool overview. In the ‘Adhesin database’ tab we provide all genes and systems with associated information collected throughout the study. In the ‘Analysis: Input & settings’ tab, users can upload their genome assemblies and run analysis. **b** When adhesiomeR analysis is finished, output tabs will appear, where users can investigate the results in the form of tables and plots. We also provide a comprehensive HTML report that can be downloaded. **c** Functions implemented in the adhesiomeR package

Two search modes, strict and relaxed (Fig. 1c), provide functionality to either identify small numbers of adhesins with high confidence or a larger number of adhesins with less confidence. The strict search mode uses gene-specific bit score thresholds, calibrated on a reference set of adhesin sequences, similar in principle to the annotation strategy implemented in CARD-RGI [21] (see Methods and Additional Files 2–3 for details). The relaxed version uses by default 80% coverage and 80% identity threshold, which may be modified by the user.

AdhesiomeR integrates search results into an overview of adhesin systems encoded across multiple genes within a gene cluster. Adhesion systems are reported as present if all genes were detected, and as partial if at least one gene was identified. Note that a system does not always correspond to a single operon. For example, some fimbriae such as curli are encoded by two differentially transcribed operons but still constitute one system [22].

The adhesiomeR web server, implemented using *shiny* R package [23] and *ShinyProxy*, allows running analyses of up to 20 genome assemblies (< 100 MB total size) in the strict search mode (Fig. 1a). It offers calculations of gene and system presence/absence as well as determination of adhesin profiles and clusters. A wide selection of charts allows for a visual inspection of results, and downloadable HTML reports can be saved for later reference and sharing of result (Fig. 1b). More customizable workflows for larger genome sets, pangenomes or gene catalogues, are possible with adhesiomeR standalone R package. This supports the analysis of a single pangenome or gene catalogue, tracing adhesion systems back to their metagenome/genome of origin. Step-by-step tutorials for the analysis of pangenomes and gene catalogues are provided in Additional Files 8–10. R package allows parallel processing using multisession option from the *future* package [24]. AdhesiomeR was developed following the FAIR Principles for research software (Table S1).

### Profiling the *E. coli* adhesiome

We developed a unified and reproducible characterization of *E. coli* adhesiomes by defining adhesin profiles and profile clusters. This follows the idea of in silico serotyping that enables determination of genes encoding combinations of surface antigens such as LPS, flagella or capsule [25]. Adhesin profiles correspond to a specific pattern of the presence/absence of adhesin genes, while adhesiome clusters represent groups of profiles with shared patterns in their adhesin repertoire that have the potential to confer characteristic phenotypes, niche adaptation or pathotype. We investigate profiles and clusters using three gene subsets: (i) A-, all adhesin genes, (ii) F-, fimbrial adhesins only, (iii) N-, nonfimbrial genes only. We propose the following classification scheme to denote adhesin profiles and clusters: profiles are denoted with numbers, whereas clusters with letters (Table 1). Those based on different gene subsets are prefixed with letters corresponding to the given subset. Therefore, A-1, F-2, and N-4 indicate profile 1 of all adhesins, profile 2 of fimbrial adhesins and profile 4 of nonfimbrial adhesins, respectively. On the other hand, A-A, F-A and N-A indicate adhesiome cluster A based on all, fimbrial and nonfimbrial adhesins, respectively (Table 1).

**Table 1:** Overview of adhesiome profiling scheme

Level of adhesin typing	Description	Gene subset	Assigned codes
Profiles	Specific pattern of adhesin gene presence/absence	All genes	From A-1 to A-7038
		Fimbrial	From F-1 to F-4770
		Nonfimbrial	From N-1 to N-1443
Clusters	General groups of profiles possessing certain characteristic features	All genes	From A-A to A-J
		Fimbrial	From F-A to F–H
		Nonfimbrial	From N-A to N-E

For each of the gene subsets, we identified all profiles amongst 15,559 pathotyped genomes. Subsequently, we sorted and numbered profiles from the most frequently occurring to the rarest (Additional Files 4–6), as well as collected general information such as the number of unique profiles with specific patterns of gene presence/absence (Table S2). In total, we identified 7,038, 4,770 and 1,443 unique profiles when considering all adhesins (A-), fimbrial adhesins (F-) and nonfimbrial adhesin genes (N-), respectively. Especially among fimbrial adhesins, some profiles were notably overrepresented, for example the F-1 profile was identified in 1,971 genomes.

Clustering of adhesin profiles revealed patterns and associations of certain adhesin genes with known *E. coli* pathotypes. Since the distribution of pathotypes in our collection of genomes was unbalanced, with the majority of genomes belonging to nonpathogenic, unknown, and UPEC, genome counts were normalized to allow comparison of pathotype content in each cluster (see Methods for more details). Representation of pathotypes in clusters before normalization is presented in Figure S3.

**Fig. 2:**
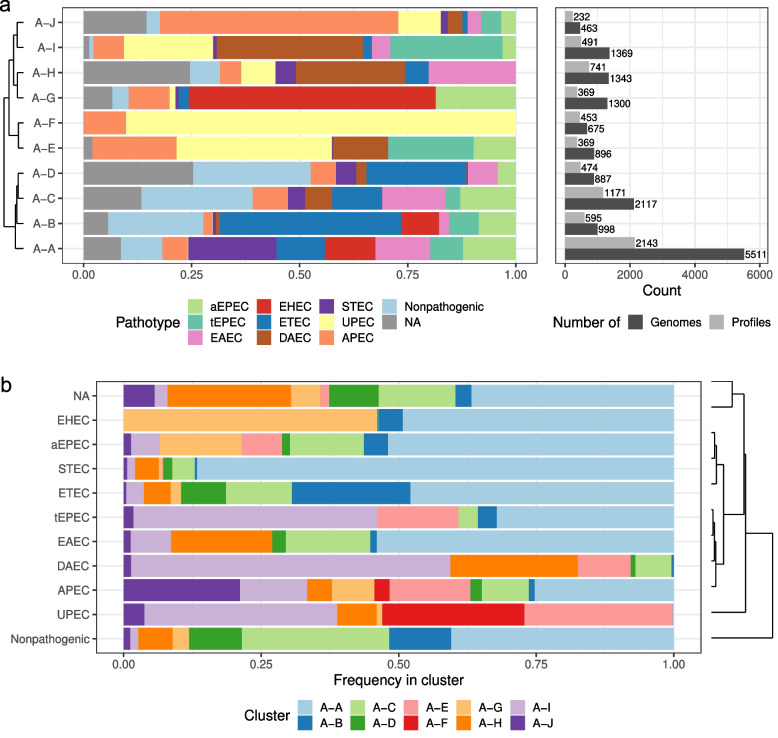
Adhesiome clusters based on all adhesins. **a** Composition of the clusters. Bar plots on the left side show the pathotype composition of each cluster with a dendrogram depicting relationships between clusters. Bar plots on the right side indicate the number of genomes and adhesin profiles that each cluster represents. **b** Assignment of pathotypes to the clusters. Bar plot shows frequency of each pathotype in the clusters

Clusters A-A, A-B, A-C, and A-D form a closely related clade with a similar distribution of pathotypes (Fig. 2a). These four clusters contain majority of unknown, EHEC, aEPEC, STEC and ETEC (Fig. 2b). Interestingly, EHEC is found in both A-A and A-B but generally not in A-C and A-D. Cluster A-A is the largest and most variable cluster comprising multiple pathotypes. It includes 86% of all STEC strains (Fig. 2b, Table 2, Fig. S4-S5) and is associated with a high prevalence of *ehaG* and Stg fimbriae (Fig. S6). A-E and A-F clusters are closely related and comprised mainly of UPEC strains (Fig. 2a). UPEC strains are generally found in these two clusters or A-I (Fig. 2b). A-I also represents a cluster to which majority of DAEC and tEPEC are classified. A-G contains mostly one subtype of EHEC, and its most distinct feature is the presence of long polar fimbriae (Fig. 2, S6). Cluster A-H comprises mainly unknown, DAEC and EAEC, and its typical feature is the presence of *aatB* autotransporter (Fig. S6). A-J is a small cluster composed mostly of APEC (Fig. 2, Table 2).

**Table 2:** Characterization of adhesin clusters

Cluster	Most prevalent pathotypes	Genes with the highest Gini importance
All adhesins		
A-A	STEC (20%), EAEC (12%), aEPEC (12%), EHEC (12%)	*ehaG*, *stgD*, *stgB*, *stgC*, *stgA*
A-B	ETEC (42%), nonpathogenic (22%)	*ecpE*, *ecpB*, *ecpC*, *ecpA*, *ecpD*
A-C	Nonpathogenic (26%), EAEC (15%), unknown (13%)	*yhcF*, *yadC*, *yhcD*, *yhcA*, *htrE*
A-D	Nonpathogenic (27%), unknown (25%)	*fimC*, *fimI*, *fimG*, *fimD*, *fimA*
A-E	UPEC (36%), tEPEC (20%)	*yfcP*, *focI2/sfaD/sfaD2*, *focC/sfaE/sfaE2*, *focF/sfaG/sfaG2*, *yfcR*
A-F	UPEC (90%)	*focC/sfaE/sfaE2*, *focF/sfaG/sfaG2*, *focB/sfaB/sfaB2*, *focD/sfaF/sfaF2*, *focI1/sfaC/sfaC2*
A-G	EHEC (57%)	*lpfC*, *lpfD*, *lpfC’*, *lpfE*, *lpfA*
A-H	DAEC (25%), unknown (25%)	*aatB*, *yraH*, *yraI*, *yraK*, *yraJ*
A-I	DAEC (34%), tEPEC (26%)	*yqiG*, *aufF*, *aufA*, *ycbF*, *ygiL*
A-J	APEC (55%)	*stgB*, *stgC*, *stgA*, *ybgO*, *ybgP*
Fimbrial adhesins		
F-A	UPEC (36%), APEC (23%)	*focC/sfaE/sfaE2*, *focI2/sfaD/sfaD2*, *focB/sfaB/sfaB2*, *focD/sfaF/sfaF2*, *uclD*
F-B	UPEC (90%)	*focI2/sfaD/sfaD2*, *focC/sfaE/sfaE2*, *focB/sfaB/sfaB2*, *focD/sfaF/sfaF2*, *focF/sfaG/sfaG2*,
F–C	STEC (20%), EAEC (13%), ETEC (12%), aEPEC (12%)	*stgD*, *stgB*, *stgC*, *stgA*, *yadV*
F-D	Nonpathogenic (24%), unknown (14%), aEPEC (13.5%)	*yhcA*, *yhcD*, *gltF*, *yhcE*, *yadC*
F-E	ETEC (47%), nonpathogenic (24%)	*fimI*, *fimA*, *ecpA*, *fimC*, *ecpC*
F-F	EHEC (65%)	l*pfA*, *lpfB*, *lpfC*, *lpfC’*, *lpfD*
F-G	DAEC (30%), tEPEC (24%)	*yfcP*, *ycbF*, *ycbV*, *yfcQ*, *ycbT*
F–H	DAEC (22%), unknown (21%), EAEC (18%)	*yraK*, *yraJ*, *yraH*, *yraI*, *sfmC*
Nonfimbrial adhesins		
N-A	EHEC (28%), aEPEC (26%)	*paa*, *eae*, *ehaG*, *ehaA*, *flu*
N-B	STEC (25%), EAEC (20%), EHEC (12%)	*iha*, *flu*, *ehaG*, *ehaA*, *cah*
N–C	Nonpathogenic (29%), ETEC (18%), unknown (16%)	*yeeJ*, *aatA*, *ypjA*, *ehaA*, *aatB*
N-D	UPEC (14%), APEC (14%), DAEC (13%), tEPEC (13%)	*ehaG*, *cah*, *yeeJ*, *aatB*, *ehaA*
N-E	APEC (25%), UPEC (25%), unknown (15%)	*tia*, *cah*, *ycgV*, *ypjA*, *aatB*

For each cluster the most prevalent pathotypes (after normalization of genome counts, see Methods) are listed and five genes with the highest Gini importance, as determined by random forest classification, regardless of importance value. Numbers in brackets indicate percentage of genomes of given pathotype in the cluster

Within the fimbrial adhesin subset, the F-A and F-B represents two closely related clusters containing mainly UPEC and APEC strains (Fig. S4b). F1C and UCL fimbriae are overrepresented in these clusters and likely indicative of UPEC pathotypes, as also reported previously [26–28]. F–C, F-D and F-E create another closely related clade. F–C contains the majority of STEC strains, and its characteristic feature is the presence of Stg fimbriae (Fig. S5-S6). A typical feature of F-D is a high prevalence of Yhc and Yad fimbriae (Fig. S6), whereas F-E is characterized by a high prevalence of ETEC (Fig. S4b, Table 2). F-F is the smallest cluster characterized mainly by EHEC strains and the presence of long polar fimbriae (Fig. S4b, S6). F-G comprise primarily DAEC, tEPEC and UPEC with Yfc and Ycb fimbriae, whereas F–H represents mainly DAEC, unknown and EAEC strains with genes encoding Yra fimbriae (Fig. S4-S6, Table 2).

Nonfimbrial adhesin clusters form two main clades. The first includes N-A and N-B, both characterized by the presence of the *ehaA* and *ehaG* genes; these clusters also contain almost all EHEC strains (Fig. S4c, S6). The most important genes in the N-A cluster are *paa* and intimin, whereas *iha* and *flu* are specific for N-B, which comprise mainly of STEC and EAEC (Fig. S4c, S6). N–C is represented mostly by nonpathogenic and unknown strains often carrying the *yeeJ*, *aatA* and *ypjA* genes (Fig. S4c, S6). N-D does not have one dominant pathotype but contains multiple pathotypes in similar ratios and is characterized by a high prevalence of *ehaG*, *cah* and *yeeJ* (Fig. S4c, S6). N-E forms the cluster specific for UPEC, APEC and uknown strains with *tia*, *cah*, *ycgV* and *ypjA* being the most characteristic genes (Fig. S4c, S6).

### Validation with experimental data

**Fig. 3:**
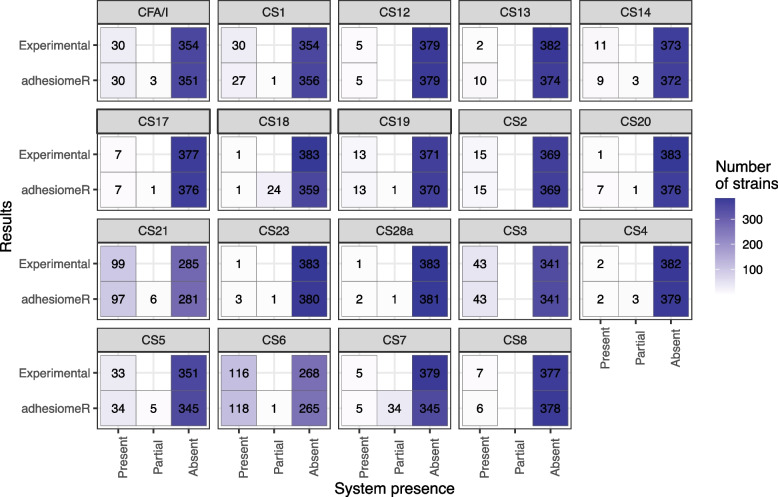
Comparison with experimental results on ETEC strains. Each subplot corresponds to one of 19 colonization factors investigated by von Mentzer et al. and shows in how many strains adhesiomeR identified given system as present, partial or absent compared to experimental analyses

Von Mentzer et al. investigated the presence of 19 colonization factors in 354 human-isolated ETEC strains using dot-blots and PCRs [29]. We used these *E. coli* genomes to evaluate accuracy of adhesiomeR compared to published experimental analyses of colonization factors. CFs are a group of adhesins commonly found in ETEC that create structures dependent on multiple subunits encoded across a gene cluster (adhesin system or operon). Our adhesiomeR results were highly consistent with experimental results obtained by von Mentzer et al. When considering partial hits to operons as absence, adhesiomeR achieved 98% accuracy and Mathews correlation coefficient 0.956. Interestingly, among 133 genomes reported as not possessing any of the investigated colonization factors (CF-negative), adhesiomeR identified at least one full or partial operon in 19 and 35 genomes, respectively (Fig. 3).

## Discussion

To date, the majority of studies have focused on the analysis of a single or a few *E. coli* adhesins and their role in pathogenicity or gut colonization [28, 30]. They provide valuable insight into the overall role of adhesins in these processes but have been limited by the lack of suitable databases and analysis tools. Our approach facilitates a holistic and standardized view of *E. coli* adhesion systems. This approach can be used to systematically investigate the role of adhesiomes in niche competition for space in the gut, at infection sites or during biofilm formation, among others.

UPEC is known as a successful colonizers of the gut and urinary tract, whereas ETECs are gastrointestinal pathogens [6, 7]. Accordingly, the adhesiome profiles and clusters we introduced reveal that ETEC and UPEC adhesin repertoires differ markedly. Moreover, adhesiome clustering shows a divergence of these pathotypes into groups with certain adhesin repertoires as they fall into multiple clusters (Fig. 2). This may indicate that adhesiomes contribute to their adaptation to specific niches.

The increasing availability of sequencing data has drastically changed the field of clinical microbiology and pathogen diagnostics. The increasing amount of data requires easy to use and interpret computational tools to enable the identification of potential microbial pathogens and the use of in silico diagnostics [31]. Many tools have been developed to allow the search for antimicrobial resistance genes and virulence factors [17, 21, 32]. However, despite their crucial role in the process of pathogenesis, adhesins still constitute only a fraction of all VAFs in currently available databases, even for model species such as *E. coli*. Therefore, a detailed characterization of adhesiomes is not possible with available tools as they often do not include multiple fimbrial adhesins. AdhesiomeR, with our collection of *E. coli* adhesins, is the first step towards a more holistic understanding of adhesiome role in host colonization. Furthermore, adhesin sequences gathered in our database can be used as a starting point for new adhesin discovery by identifying protein domains characteristic to adhesins or predicted structural similarity [33, 34]. Such systematic approach could be extended to search for new adhesins in other priority pathogens with high clinical significance.

The high level of agreement between adhesiomeR results and experimental studies (98% overlap) demonstrates the applicability of our tool to genomic studies of *E. coli* strains and metagenomes, to investigate adhesiomes at high-confidence and without the need for experimental validation. Moreover, adhesiomeR identified full operons encoding colonization factors in genomes annotated as CF-negative based on experimental analyses, showing its high sensitivity. These differences between in silico and experimental analyses might be explained by known problems associated with PCR and dot-blot analyses. Negative results of the PCR may be caused by mutations in fragments targeted by the primers [35], whereas dot-blot analyses would not identify fimbriae that are currently not being expressed. On the other hand, the presence of the full operon indicated by adhesiomeR is very unlikely to be a result of false positives as it requires hits to all genes across the system, which usually comprises at least four genes. The gene-specific thresholds additionally restrict results to high-confidence hits. Therefore, a more probable explanation for observed divergent results is lower sensitivity of experimental procedures. Overall, this comparison showed that adhesiomeR offers a convenient way of detailed *E. coli* adhesiome characterization and might provide higher sensitivity in the identification of adhesins compared to experimental procedures.

## Conclusions

The worldwide rise of antimicrobial-resistant *E. coli* as well as the increase in detection of non-communicable diseases, such as IBD in developed countries, require new strategies to characterize AMR and disease-causing *E. coli* in humans and animals. AdhesiomeR fills the gap in the field of virulence-associated factors by focusing specifically on adhesins and offers cost-effective determination of *E. coli* adhesiomes. We propose a systematic scheme for profiling of *E. coli* adhesiomes using adhesin profiles and adhesiome clusters obtained with our software. Classification into adhesin profiles and adhesiome clusters provides insights into the adhesion and pathogenicity of analysed strains. Results obtained with adhesiomeR can assist experimentalists in characterizing *E. coli* strains relevant to human and animal health and guide the development of novel treatment strategies against AMR and pathogenic *E. coli*. The use of adhesiomeR for analysis of epidemiological *E. coli* strains could also influence the vaccine target selection leading to a better outbreak prevention.

## Methods

### Data acquisition

To collect all available sequences of *E. coli* adhesins, a literature search of adhesin reviews and original studies describing specific adhesins was conducted. Where possible, we used the sequences referenced in papers (93% gene sequences), otherwise we searched for sequences in the GenBank database. As a supplement to sequences, we also collected information about the function or class of each gene, i.e., if it encodes a structural subunit, tip adhesin, usher or chaperone in case of fimbrial adhesins (available on the adhesiomeR web server). In total, we collected 525 genes encoding adhesins grouped into 102 systems.

We downloaded 25,436 genomes of *E. coli* and associated metadata available in RefSeq on 26.10.2021. We filtered out genomes with (i) coverage < 50 and contig N50 < 1,000,000, (ii) sequenced only using 3rd generation platforms, (iii) sequencing platform not specified and contig N50 < 1,000,000, (iv) containing > 400 contigs. We performed in silico pathotyping, i.e. differentiating into groups with a certain pathogenicity and virulence factors, of obtained genomes into aEPEC, DAEC, EAEC, EHEC, EIEC, ETEC, NMEC, STEC, tEPEC, UPEC, nonpathogenic and unknown (NA). This procedure was based on the presence and/or absence of characteristic virulence factors of these pathotypes (Table S3-4) [18, 19]. Additionally, we included 354 ETEC genomes from a paper by Von Mentzer et al. [29] and 573 APEC genomes from multiple studies (Table S5). The final collection consisted of 15,559 genomes (Additional File 7).

For construction of exemplary pangenome, we used 72 strains from the *E. coli* reference collection (ECOR) [36]. To ensure compatibility of output formats, we built pangenome using two popular algorithms, Roary [37] and panaroo [38]. As an example of a metagenomic dataset, we used data from a study by Hildebrand et al. [39].

### Selection of gene-specific bit score thresholds

To obtain the reference set, we first analysed the genome collection with adhesiomeR in the relaxed setting using 80% identity and 80% coverage cut-offs. Next, we analysed fimbrial and nonfimbrial adhesins separately. For the former, we selected results with complete systems localized on a single contig and plotted the gene localization to visually investigate the quality of the results. Using this approach, we were able to further exclude results, where genes in the system were not located next to each other in correct order or containing transposon insertions. If for a certain system we found less than 15 occurrences of a complete system on a single contig, we also considered results where genes of that system were found on multiple contigs. The set of results with intact systems was considered a reference set for fimbrial adhesins. In the case of nonfimbrial adhesins, which are encoded by single genes, we investigated their genomic context by searching for two genes located upstream and downstream of the adhesin-encoding gene. If the hits were in the same genomic context, we considered them as reference hits. Based on these results, we set a gene-specific bit score threshold by selecting the minimum value of bit score for a given gene in the reference set (Additional File 2). For some adhesins, we were unable to find a set of reference sequences as they were found only in a few cases or not at all. For 22 genes (draA, drab, draC, draD, draE, fotC, fotD, fotE, fotF, fotG, cs27a_gene1, cs27a_gene2, cs27a_gene3, cs27a_gene5, cs27a_gene6, cs27a_gene7, cs27a_gene8, cs27a_gene9, cs27b_gene1, cs27b_gene3, cs27b_gene4, cs27b_gene5, cs27b_gene6, cs27b_gene7) we estimated bit score thresholds based on genes of the same/very similar length from homologous operons. For 11 adhesin genes (afrA, afrB, afrC, afrD, afrE, afrR, afrS, fotA, fotB, cs27a_gene4, cs27b_gene2) which do not show homology to subunits from other systems, we estimated bit score thresholds based on genes of similar length.

### Clustering of adhesin profiles

We used unique adhesin profiles of adhesin gene presence/absence to create adhesiome clusters. For this procedure, only genes found in at least one genome assembly were used. For each gene subset an optimal number of clusters was determined using gap statistic. The clustering was performed using Manhattan distance and clara algorithm [40].

To determine the most important adhesins for each cluster, a random forest classifier was trained on each of the three gene subsets of the data, using the ranger R package with default parameters [41]. Feature importance was calculated to see which genes contribute the most to the prediction of a certain cluster and finally 20 genes with the highest Gini importance were selected within each cluster (Fig. S6).

Our collection of *E. coli* genomes was highly unbalanced. Strains classified as nonpathogenic, unknown, and UPEC were overrepresented, comprising 33.7%, 18.9% and 16.3% of the whole collection, respectively. The least represented pathotypes in our collection were NMEC (9 strains, 0.06% of all genomes) and EIEC (30 strains, 0.19% of all genomes), which have been removed for visualisations of cluster compositions. To allow meaningful analysis of pathotype composition of the clusters, the numbers of genomes were normalized to make them comparable. The cluster composition before normalization is available in Fig. S3.

### Performance evaluation

Von Mentzer et al. [29] investigated the presence of 19 colonization factors in human-isolated ETEC strains using dot-blots and PCRs. The whole genome sequence of 354 strains from their study were used to evaluate if adhesiomeR correctly identified CF systems investigated previously. To do that, adhesiomeR was run using strict version of the search and obtained system presence was compared with experimental results. For calculation of accuracy and MCC, only presence of the full operon was considered as a positive identification of a system, whereas ‘partial’ hits were treated as negative result and combined with ‘absent’. Accuracy was calculated as percent of correct assignments $\frac{TP+TN}{TP+FP+TN+FN}\times 100\%$, where TP – true positives, TN – true negatives, FP – false positives, FN – false negatives. MCC was calculated as $\frac{TP\times TN-FP\times FN}{\sqrt{(TP+FP)\times (TP+FN)\times (TN+FP)\times (TN+FN)}}$.

### Supplementary Information

Additional file 1. Figures S1-S6 and Tables S1-S5.

Additional file 2: Table A1. List of reference hits used to set gene-specific bit score thresholds.

Additional file 3: Table A2. Adhesin gene-specific bit score thresholds. These thresholds are used to determine gene presence absence in the strict version of the search.

Additional file 4: Table A3. Adhesin profiles based on all adhesin genes. The first column indicates the profile number, whereas the remaining columns list genes determining the profiles and their presence (1) or absence (0) in each profile.

Additional file 5: Table A4. Adhesin profiles based on fimbrial adhesin genes. The first column indicates the profile number, whereas the remaining columns list genes determining the profiles and their presence (1) or absence (0) in each profile.

Additional file 6: Table A5. Adhesin profiles based on nonfimbrial adhesin genes. The first column indicates the profile number, whereas the remaining columns list genes determining the profiles and their presence (1) or absence (0) in each profile.

Additional file 7: Table A6. List of *in silico* pathotyped genomes.

Additional file 8: Tutorial_genome_assemblies. Tutorial describing how to run adhesiomeR analysis of genome assemblies using R package.

Additional file 9: Tutorial_pangenomes. Tutorial describing how to run adhesiomeR analysis of a pangenome using R package.

Additional file 10: Tutorial_metagenomics. Tutorial describing how to run adhesiomeR analysis of metagenomic gene catalogue using R package.

## Data Availability

E. coli genomes used in the study, including APEC and human-isolated ETEC, are available from NCBI Assembly (https://www.ncbi.nlm.nih.gov/assembly) database using accession numbers listed in Additional File 7. E. coli genomes used to create pangenomes are available from ECOR [32]. Metagenomic dataset was obtained from Hildebrand et al. [35]. All code used for data analysis is available at https://github.com/ksidorczuk/adhesiomeR-paper. AdhesiomeR is available as a web server at https://adhesiomer.quadram.ac.uk/ and as an R package from GitHub https://github.com/ksidorczuk/adhesiomeR.
