# Supplementary material for: adhesiomeR: a tool for Escherichia coli adhesin classification and analysis
Source: BMC Genomics. 2024 Jun 17;25:609. doi: 10.1186/s12864-024-10525-6 (PMC11184843; doi:10.1186/s12864-024-10525-6)
Supplement: Supplementary file 1 — Additional file 1. Figures S1-S6 and Tables S1-S5. [file 12864_2024_10525_MOESM1_ESM.pdf]

Supplementary Information

adhesiomeR: a tool for *Escherichia coli* adhesin classification and analysis

Katarzyna Sidorczuk[1,2,3,4], Michał Burdukiewicz[5,6], Klara Cerk[1,2], Joachim Fritscher[1,2], Robert A Kingsley[1,7], Peter Schierack[4], Falk Hildebrand[1,2,*], Rafał Kolenda[8,1,*]

[1]Quadram Institute Biosciences, Norwich Research Park, Norwich, UK,

[2]Earlham Institute, Norwich Research Park, Norwich, UK,

[3]Department of Bioinformatics and Genomics, Faculty of Biotechnology, University of Wrocław, Poland,

[4]Institute for Biotechnology, Brandenburg University of Technology (BTU) Cottbus-Senftenberg, Senftenberg, Germany,

[5]Clinical Research Centre, Medical University of Białystok, Poland,

[6]Institute of Biotechnology and Biomedicine, Autonomous University of Barcelona, Cerdanyola del Vallès, Spain,

[7]Depertment of Biological Sciences, University of East Anglia, Norwich, UK

[8]Department of Biochemistry and Molecular Biology, Faculty of Veterinary Medicine, Wrocław University of Environmental and Life Sciences, Poland.

[*]To whom correspondence should be addressed.

Contents

1. Figures 2

   Figure S1. Data preprocessing. 2

   Figure S2. Gene localization check. 2

   Figure S3. Adhesiome clusters based on all adhesins (a), fimbrial adhesins (b), and nonfimbrial adhesins (c) before normalization of genome numbers associated with pathotypes. 3

   Figure S4. Adhesiome clusters based on all adhesins (a), fimbrial adhesins (b), and nonfimbrial adhesins (c) after normalization of genome numbers associated with pathotypes. 4

   Figure S5. Assignment of pathotypes to clusters. 5

   Figure S6. Importance of adhesin genes on adhesiome clusters. 6

2. Tables 7

   Table S1. Design of adhesiomeR in the view of FAIR4RS principles. 7

   Table S2. Summary of profiles for each gene subset. 7

   Table S3. Accessions of genes used for *in silico* pathotyping. 8

   Table S4. Criteria used for *in silico* pathotyping. 9

   Table S5. Accessions of APEC assemblies. 10

1. Figures

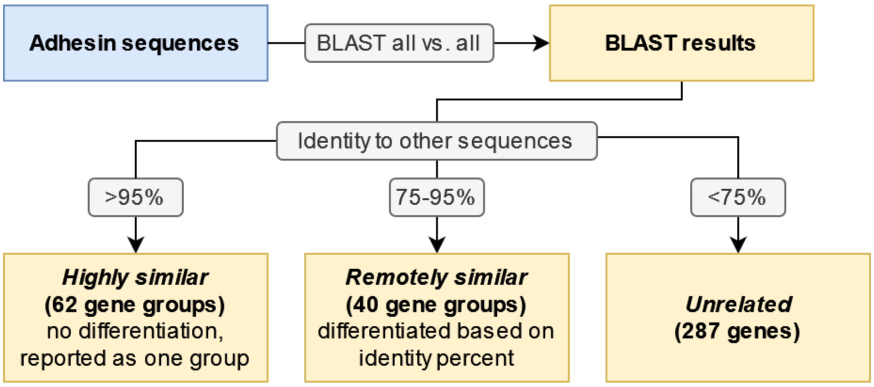

Figure S1. Data preprocessing.

We divided all sequences of adhesins we collected into three groups depending on the level of their similarity to each other. We performed BLAST all vs. all search and depending on the identity percent, we sorted sequences into following groups: (i) *highly similar*, in which we group together genes with identity to each other higher than 95%, (ii) *remotely similar*, which are similar to each other but with lower identity percent (75-95%) and (iii) *unrelated*, which do not show significant similarity to other sequences. *Highly similar* genes are reported in the results as one group, e.g. afaA/afaA-III/daaA/draA. The differentiation between *remotely similar* gene groups, e.g. pixC and sfpC or cooA and cosA, is based on identity percent, meaning that a hit from BLAST will be classified as a gene, with which it shares the highest identity percent. *Unrelated* genes do not require additional steps and are processed without additional steps.

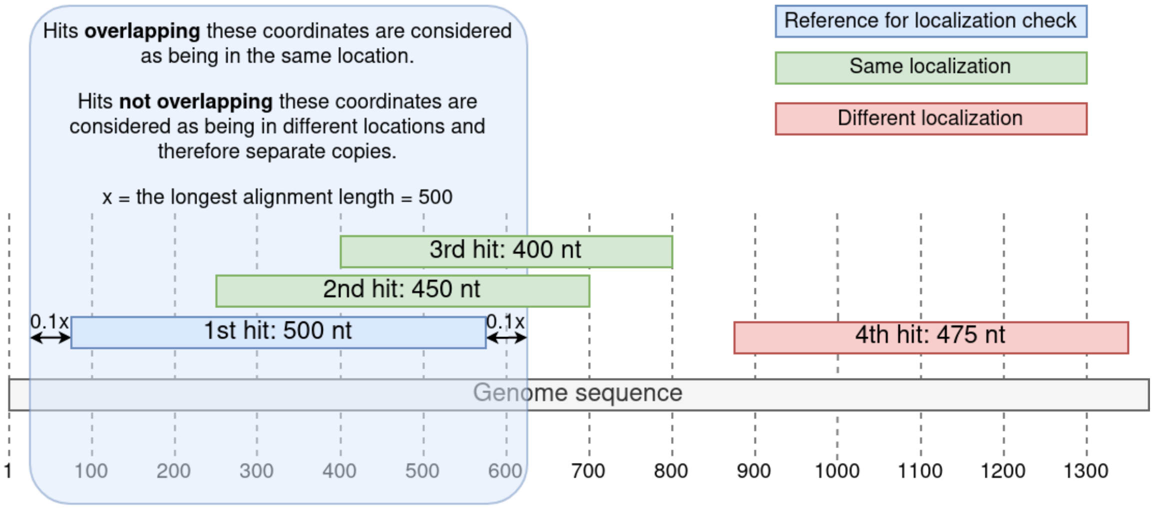

Figure S2. Gene localization check.

To perform a localization check, we first identify the longest alignment of the overlapping hits. Based on the longest alignment, we extend its start and end positions by 10% of the alignment length. If other hits overlap with this location, we treat them as having the same localization and thus the same gene.

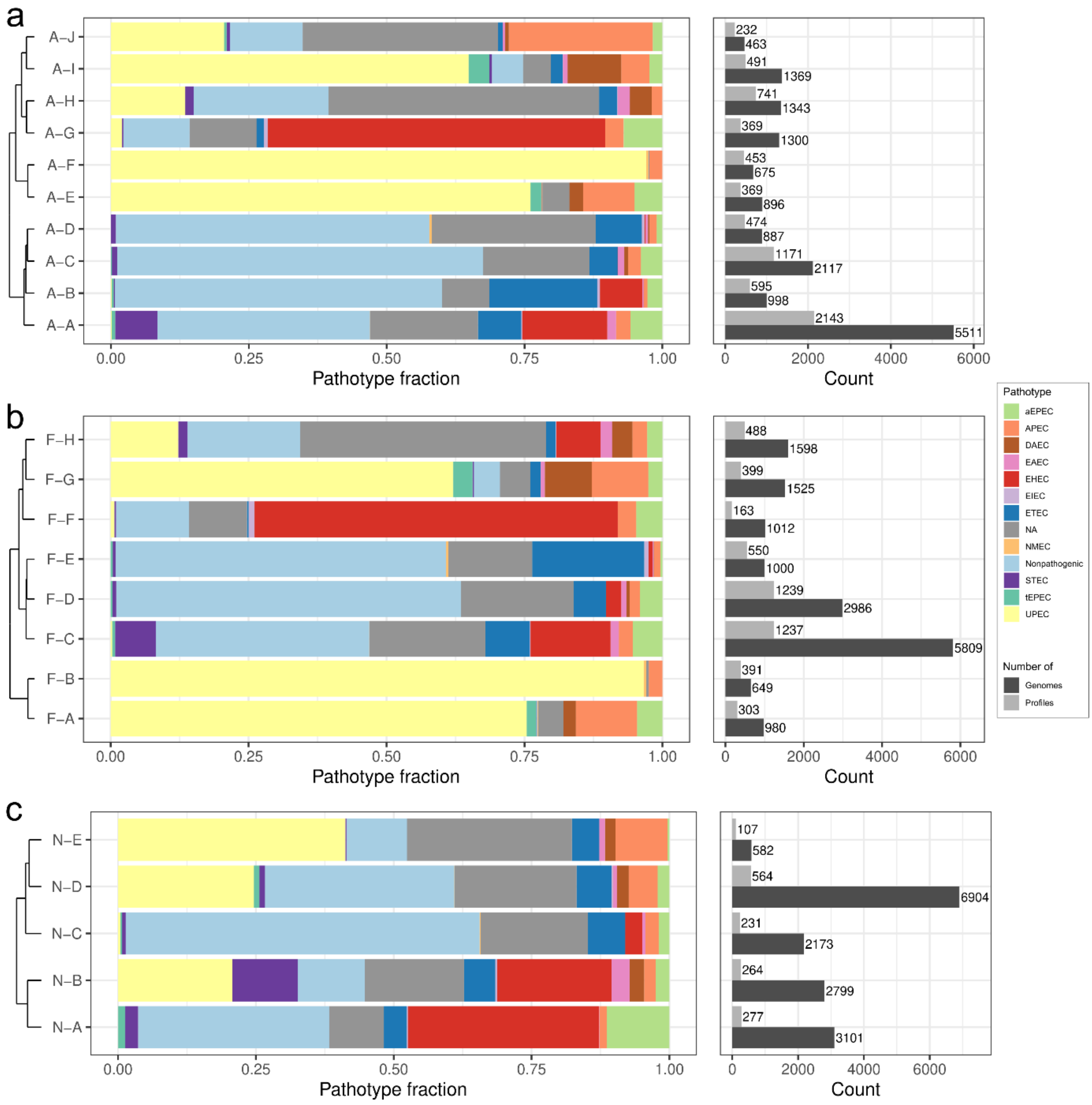

Figure S3. Adhesiome clusters based on all adhesins (a), fimbrial adhesins (b), and nonfimbrial adhesins (c) before normalization of genome numbers associated with pathotypes.

Bar plots on the left side show the pathotype composition of each cluster with a dendrogram depicting relationships between clusters. Bar plots on the right side indicate the number of genomes and adhesin profiles that each cluster represents. Pathotype fraction corresponds to fraction of genomes before normalization.

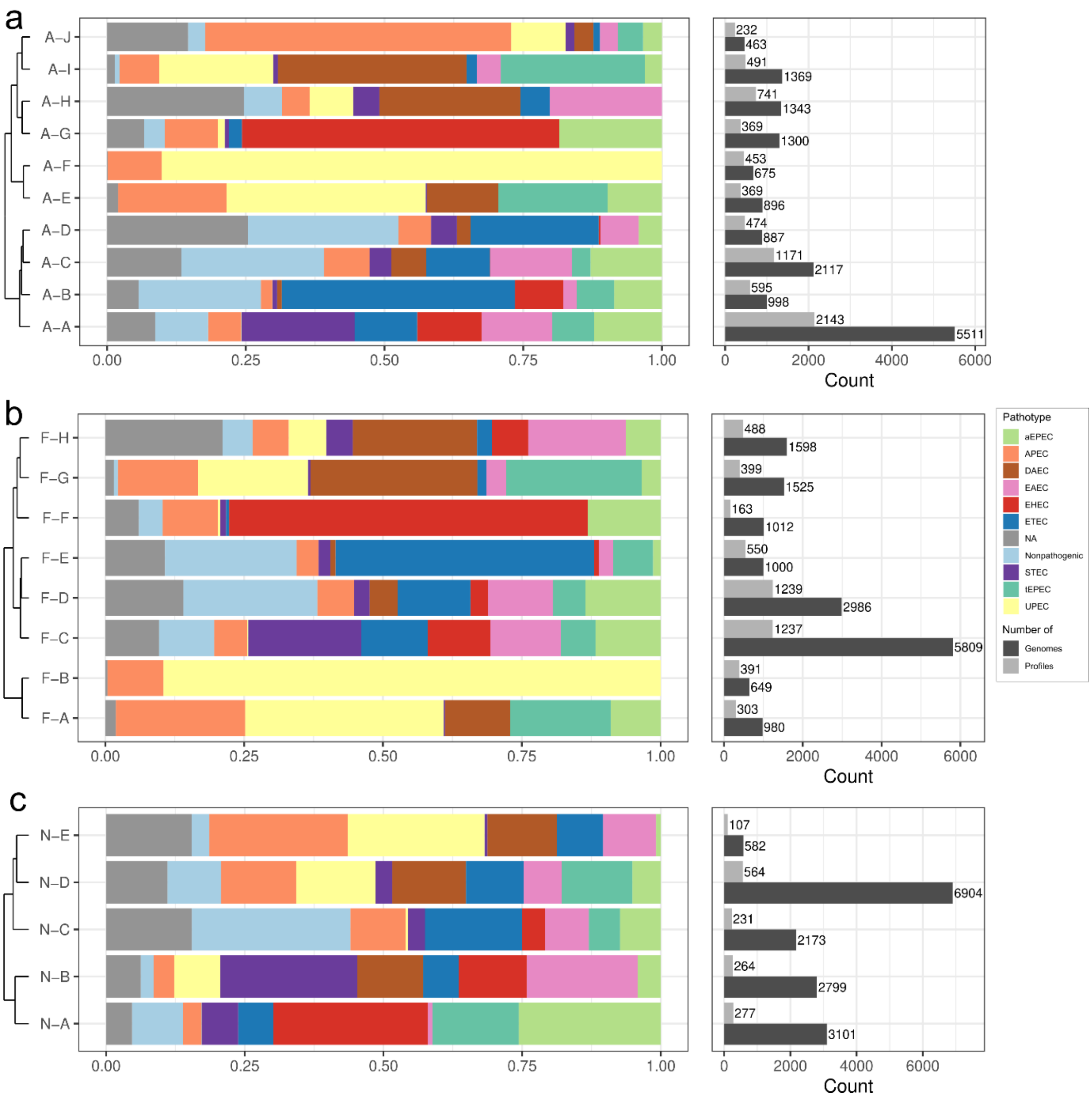

Figure S4. Adhesiome clusters based on all adhesins (a), fimbrial adhesins (b), and nonfimbrial adhesins (c) after normalization of genome numbers associated with pathotypes.

Bar plots on the left side show the pathotype composition of each cluster with a dendrogram depicting relationships between clusters. Bar plots on the right side indicate the number of genomes and adhesin profiles that each cluster represents. Pathotype fraction corresponds to fraction of genomes after normalization. NMEC and EIEC have been removed from the pathotype composition plot due to the very low number of genomes in the collection.

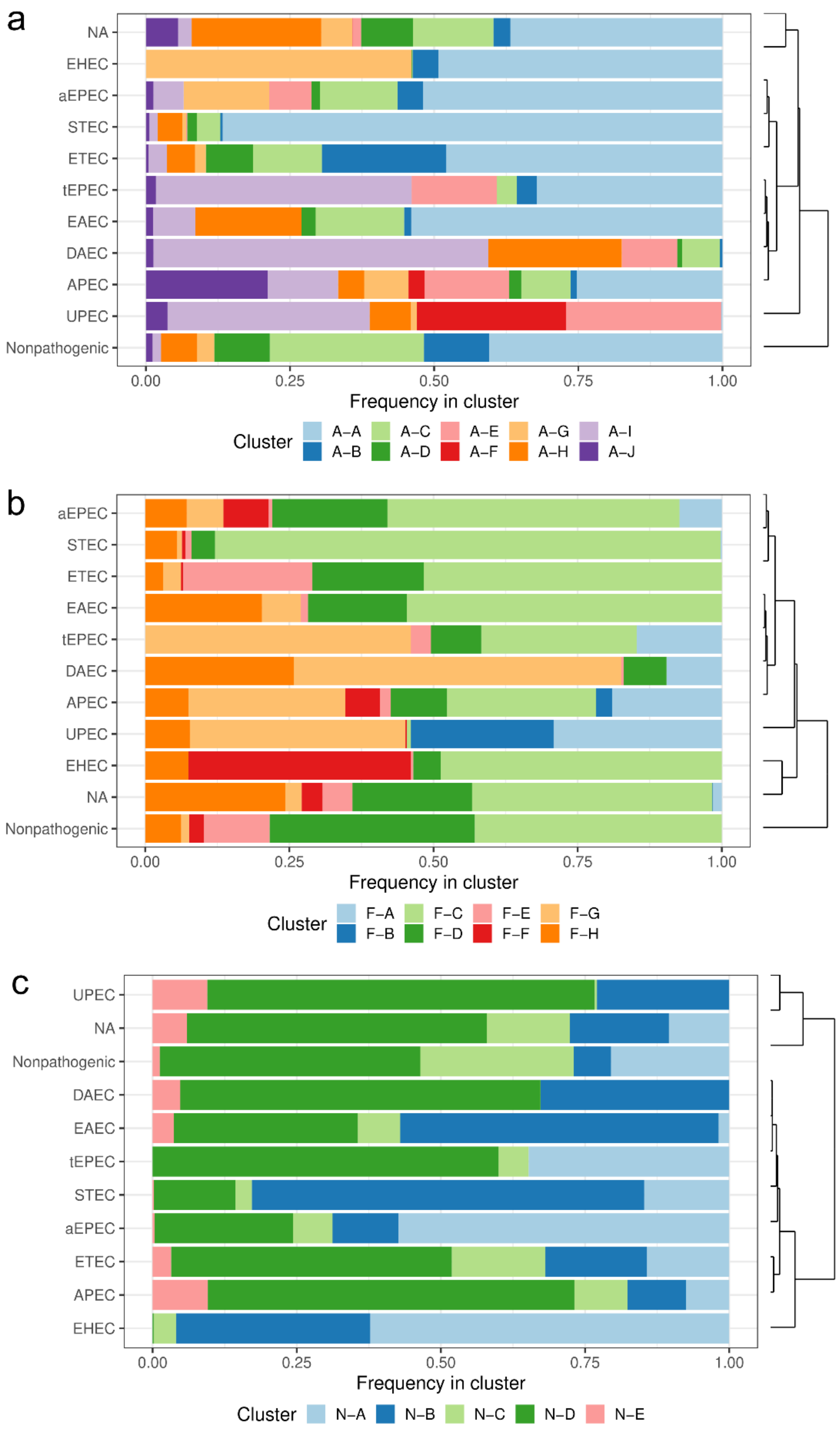

Figure S5. Assignment of pathotypes to clusters.

Bar plots show distribution of pathotypes among clusters based on all adhesins (a), fimbrial adhesins (b) and nonfimbrial adhesins (c). NMEC and EIEC have been removed from the pathotype composition plot due to the very low number of genomes in the collection.

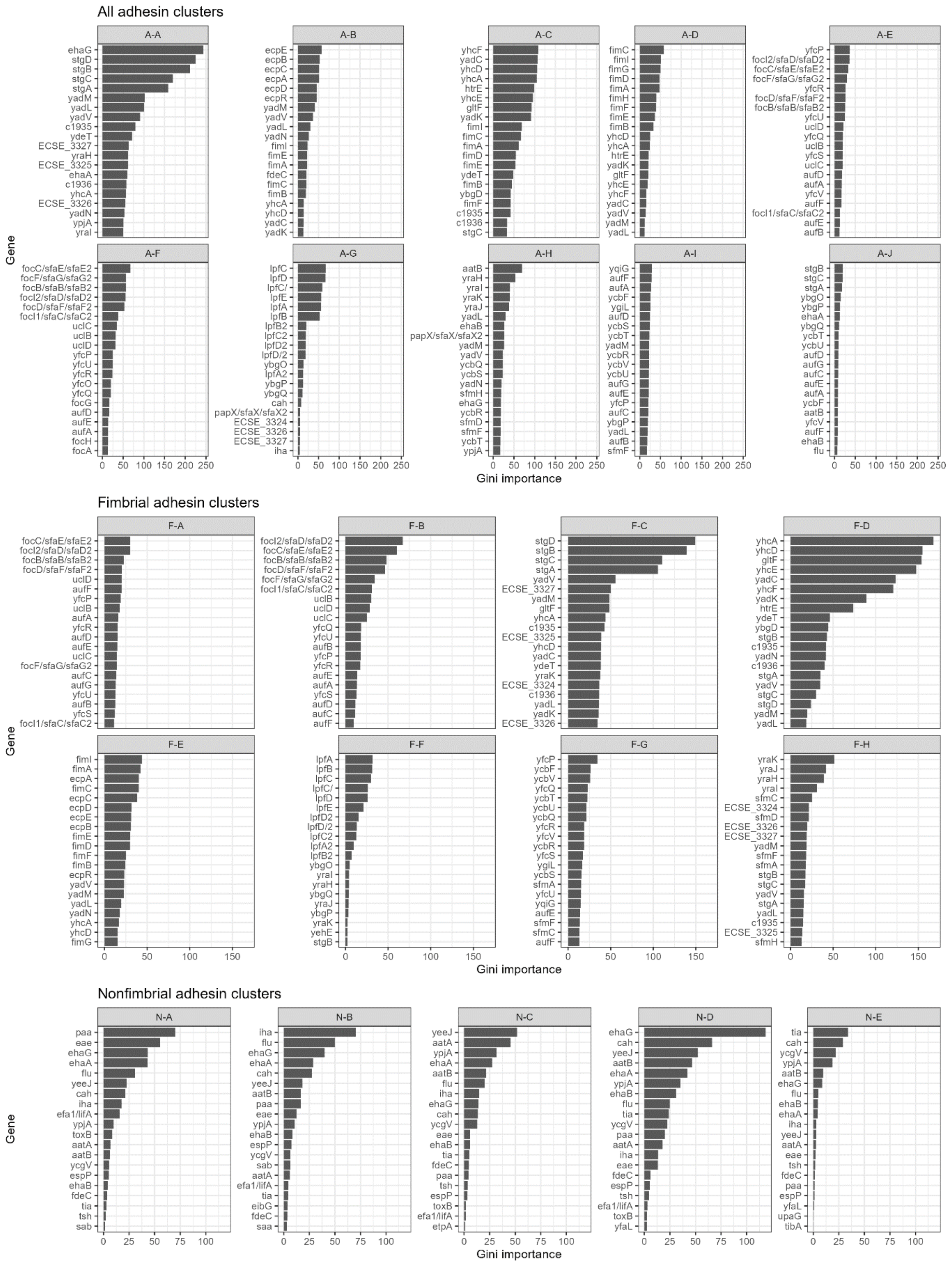

Figure S6. Importance of adhesin genes on adhesiome clusters.

Importance was determined by training a random forest model using default parameters to differentiate between one cluster and all the others. The procedure was repeated for each cluster from each gene subset. The figure shows 20 genes with the highest Gini importance for differentiation of specific cluster from the others.

2. Tables

Table S1. Design of adhesiomeR in the view of FAIR4RS principles. IDs of FAIR4RS principles are derived from Barker et al. 2023.

F1. Software is assigned a globally unique and persistent identifier.	The release versions of adhesiomeR have their individual DOIs.
A1. Software is retrievable by its identifier using a standardised communications protocol.	adhesiomeR can be downloaded from the versioned project repository.
I1. Software reads, writes, and exchanges data in a way that meets domain-relevant community standards.	The format of the input data is either a community standard (FASTA format) or is produced by well-known domain-relevant software (Roary or panaroo).
I2. Software includes qualified references to other objects.	DOIs provide the reference to the source information of data included in the adhesiomeR database.
R1. Software is both usable (can be executed) and reusable (can be understood, modified, built upon, or incorporated into other software).	adhesiomeR is implemented as the R package with the open source code. The source code of the adhesiomeR web server is a part of the R package.
R2. Software includes qualified references to other software.	BLAST, utilized by adhesiomeR, is referenced in the user manual.
R3. Software meets domain-relevant community standards.	adhesiomeR is documented using the Roxygen2 standard and written according to the principle of tidy-data.

Table S2. Summary of profiles for each gene subset. Number of profiles indicates how many distinct profiles, i.e. patterns of specific adhesin gene presence/absence, were identified in our *E. coli* genome collection. Table also shows in how many genomes the most common profile was found as well as how many genomes had profiles not observed in any other genome.

Gene subset	Number of profiles	Number of genomes with the most common profile	Number of genomes with profiles seen only in a single genome
All adhesins	7038	321	5190
Fimbrial	3414	1971	4770
Nonfimbrial	1443	574	606

Table S3. Accessions of genes used for *in silico* pathotyping.

Gene	Accession
aatA	AY351860.1:2552-3790
afaA	FM955458.1:17-322
afaE-I	FM955458.1:5290-5775
afaE-III	X76688.1:8646-9128
aggR	NC_019000.1:48472-49269
bfpA	NC_010862.1:2646-3227
chuA	NC_011750.1:c4160640-4158658
daaE	M27725.1
draE	AF329316.1:5805-6287
eae	NC_002695.1:c4599262-4596458
eltA	NC_013507.1:c22495-21719
eltB	NC_013507.1:c21722-21348
fimH	NC_011750.1:4996261-4997163
fyuA	NC_011750.1:c1134590-1132569
ial	AF348706.1:118694-119761
ipaH	M32063.1
iucC	NC_007675.1:100818-102560
neuA	NC_011750.1:c3596071-3594815
neuC	NC_011750.1:c3594818-3593643
sat	NC_004431.1:c3460261-3456362
sitA	NC_017659.1:c126422-125481
sta1	NZ_CP024249.1:c123053-122835
sta2	NC_017724.1:c35945-35727
stb	NC_018998.1:c5269-5054
stx1a	NC_002695.1:c2925716-2924769
stx1b	NC_002695.1:c2924759-2924490
stx2a	NC_002695.1:1266965-1267924
stx2b	NC_002695.1:1267936-1268205
vat	NC_008563.1:c313072-308942
yfcV	NC_011750.1:c2577809-2577243

Table S4. Criteria used for *in silico* pathotyping.

Pathotype	Full pathotype name	Genes present	Genes absent
tEPEC	Typical enteropathogenic *E. coli*	*eae*, *bfp*	*stx1a*, *stx1b*, *stx2a*, *stx2b*
aEPEC	Atypical enteropathogenic *E. coli*	*eae*	*bfp*, *stx1a*, *stx1b*, *stx2a*, *stx2b*
STEC	Shiga toxin-producing *E. coli*	*stx1a*, *stx1b* or *stx2a*, *stx2b*	*eae*, *ial*, *ipaH*, *eltA*, *eltB*, *sta1*, sta2, stb *aggR*, *afaA*, *afaE-I*, *afaE-III*, *daaE*, *draE*, *sat*, *vat*
EHEC	Enterohemorrhagic *E. coli*	*stx1a*, *stx1b*, *eae*<br>or<br>*stx2a*, *stx2b*, *eae*<br>or<br>*stx1a*, *stx1b*, *stx2a*, *stx2b*, *eae*	
EAEC	Enteroaggregative *E. coli*	*aggR*, *aatA*	
ETEC	Enterotoxigenic *E. coli*	*eltA* or *eltB* or *sta1* or *sta2*	*aggR*
EIEC	Enteroinvasive *E. coli*	*ial* or *ipaH*	*stx1a*, stx1b, stx2a, stx2b
DAEC	Diffusely adherent *E. coli*	*afaA*, *sat*, *afaE-I*<br>or<br>*afaA*, *sat*, *afaE-III*<br>or<br>*afaA*, *sat*, *daaE*<br>or<br>*afaA*, *sat*, *draE*	
UPEC	Uropathogenic *E. coli*	*fyuA*, *fimH*, *chuA*, *yfcV*<br>or<br>*fyuA*, *fimH*, *chuA*, *vat*<br>or<br>*fyuA*, *fimH*, *yfcV*, *vat*	
NMEC	Neonatal meningitis *E. coli*	*sitA*, *vat*, *neuC*, *iucC*, *neuA*	
Nonpathogenic		None or *fyuA* or *iucC* or *neuC* or *sitA* or *yfcV*	*aap*, *aatA*, *aatC*, *aatP*, *afaA*, *afaB*, *afaC*, *afaD*, *afaE-I*, *afaE-III*, *aggR*, *bfpA*, *daaE*, *draE*, *eae*, *eltA*, *eltB*, *ial*, *ipaH*, *pet*, *sat*, *sta1*, *sta2*, *stb*, *stx1a*, *stx1b*, *stx2a*, *stx2b*, *vat*

Table S5. Accessions of APEC assemblies. An asterisk indicates that number of downloaded genomes differs from number of genomes listed in the BioProject due to the unsuccessful download (2 genomes from PRJNA479542 and 2 genomes from PRJEB11876).

Accession	Database	Type of data	Downloaded genomes
PRJNA592536	BioProject	raw	259
PRJNA488670	BioProject	assembled	7
PRJNA479542	BioProject	assembled	95*
PRJNA319144	BioProject	assembled	15
PRJNA507325	BioProject	assembled	62
PRJNA553636	BioProject	raw	32
PRJEB11876	BioProject	raw	93*
CP006834	GenBank	assembled	10
CP030791	GenBank	assembled	1
CP030792	GenBank	assembled	1
CP030793	GenBank	assembled	1
CP006830	GenBank	assembled	1
CP005930	GenBank	assembled	1
CP005931	GenBank	assembled	1
CP005932	GenBank	assembled	1
HE962388	GenBank	assembled	1
HE964769	GenBank	assembled	1
AOGM00000000	GenBank	assembled	1
AOGN00000000	GenBank	assembled	1
AOGL00000000	GenBank	assembled	1
CP004009	GenBank	assembled	1
NC_008563	GenBank	assembled	1
		Total:	573
