# Supplementary material for: adhesiomeR: a tool for Escherichia coli adhesin classification and analysis
Source: BMC Genomics. 2024 Jun 17;25:609. doi: 10.1186/s12864-024-10525-6 (PMC11184843; doi:10.1186/s12864-024-10525-6)
Supplement: Supplementary file 8 — Additional file 8: Tutorial_genome_assemblies. Tutorial describing how to run adhesiomeR analysis of genome assemblies using R package. [file 12864_2024_10525_MOESM8_ESM.pdf]

Analysis of genome assemblies with adhesiomeR

Contents

Download genomes . . . 1
Run adhesiomeR analysis - strict version . . . 2
Analysis on gene level . . . 2
Plotting results on gene level . . . 3
Analysis on system level . . . 5
Plotting results on system level . . . 5
Profile analysis . . . 8
Cluster analysis . . . 9
Report generation . . . 9
Run adhesiomeR analysis - relaxed version . . . 10
Analysis on gene level . . . 10
Plotting results on gene level . . . 11
Analysis on system level . . . 13
Plotting results on system level . . . 13
Report generation . . . 16

Download genomes

This step is optional. If you already have your own genome fasta files you would like to analyse with adhesiomeR, proceed to the next step. If you do not have your own assemblies and want to try adhesiomeR on example data, please follow commands below to download 72 *Escherichia coli* reference collcetion (ECOR) strains.

```
# rentrez package is required for downloading data
if (!require("rentrez")) install.packages("rentrez")
if (!require("R.utils")) install.packages("R.utils")
library(rentrez)
library(R.utils)
library(dplyr)
library(adhesiomeR)
library(kableExtra)

# get genome accessions from data file
ecor_csv <- system.file("ecor_data.csv", package = "adhesiomeR")
ecor_data <- read.csv(ecor_csv, check.names = FALSE)
```

```
ecor_accessions <- ecor_data[["GenBank accession no."]]

# create directory for storing downloaded genomes
if(!dir.exists("./ecor_genomes/")) dir.create("./ecor_genomes/")

# download ECOR genomes in fasta format
# please be patient - it may take a few minutes
for(ith_acc in ecor_accessions) {
  search_id <- entrez_search(db = "nuccore", term = ith_acc)[["ids"]][1]
  link_to_assembly <- entrez_link(dbfrom = "nuccore",
                                  db = "assembly",
                                  id = search_id)[["links"]][["nuccore_assembly"]]
  ftp_link <- entrez_summary(db = "assembly",
                             id = link_to_assembly)[["ftppath_refseq"]]
  assembly_accession <- entrez_summary(db = "assembly",
                                       id = link_to_assembly)[["assemblyaccession"]]
  full_link <- paste0(ftp_link, "/", last(strsplit(ftp_link, "/")[[1]]),
                      "_genomic.fna.gz")
  file_name <- last(strsplit(full_link, "/")[[1]])
  download.file(url = full_link, destfile = paste0("./ecor_genomes/", file_name))
  gunzip(paste0("./ecor_genomes/", file_name))
  print(paste0("Downloaded ", assembly_accession))
}
```

Run adhesiomeR analysis - strict version

If you have the files ready, you can run adhesiomeR analysis to search for known adhesins.

Analysis on gene level

First, you need a vector of file names you wish to analyse. To obtain it for genomes downloaded in the previous step, run:

```
genomes <- list.files("./ecor_genomes/", full.names = TRUE)
```

Now, you can run blast search and specify number of threads to use with `n_threads` argument.

```
library(adhesiomeR)
blast_results <- get_blast_res(genomes, n_threads = 4)
```

BLAST analysis will create some temporary output files that are deleted after successful BLAST run. By default, they are placed in the current working directory but you can change it using `tmp_dir` argument. Note that it should be an existing directory to which you have write access.

```
blast_results <- get_blast_res(genomes, n_threads = 4, tmp_dir = "./tmp")
```

The next step is to get gene presence information from blast results. Here, 1 indicates gene presence and 0 its absence.

```
presence_df <- get_presence_table_strict(blast_res = blast_results,
                                         n_threads = 8)
```

You can save the table to a csv file:

```
write.csv(presence_df, file = "Your_filename.csv", row.names = FALSE)
```

If you wish to see only genes that were found in at least one file, you can set `add_missing` argument to `FALSE`. Note that by default the results include all genes from the adhesiomeR database.

```
presence_df2 <- get_presence_table_strict(blast_res = blast_results,
                                          n_threads = 8,
                                          add_missing = FALSE)
```

If you are interested in number of gene copies, you can use `count_copies` argument:

```
copies_df <- get_presence_table_strict(blast_res = blast_results,
                                       n_threads = 8,
                                       count_copies = TRUE)
```

Plotting results on gene level

You can easily plot the presence/absence of adhesin genes. For simplicity (and due to the size of the full plot), we will plot only a few systems: type 1, Auf, Yhc, Pix, UCL fimbriae, ehaB, cah and paa. By default (without specifying `systems` argument), genes from all systems will be plotted. Note that if you analyse more than one genome, the results on a heatmap are clustered for more clear visualisation.

```
get_presence_plot(presence_table = presence_df,
                  systems = c("Type_1", "Auf", "Yhc", "Curli",
                              "P_1", "UCL", "ehaB", "cah", "paa"))
```

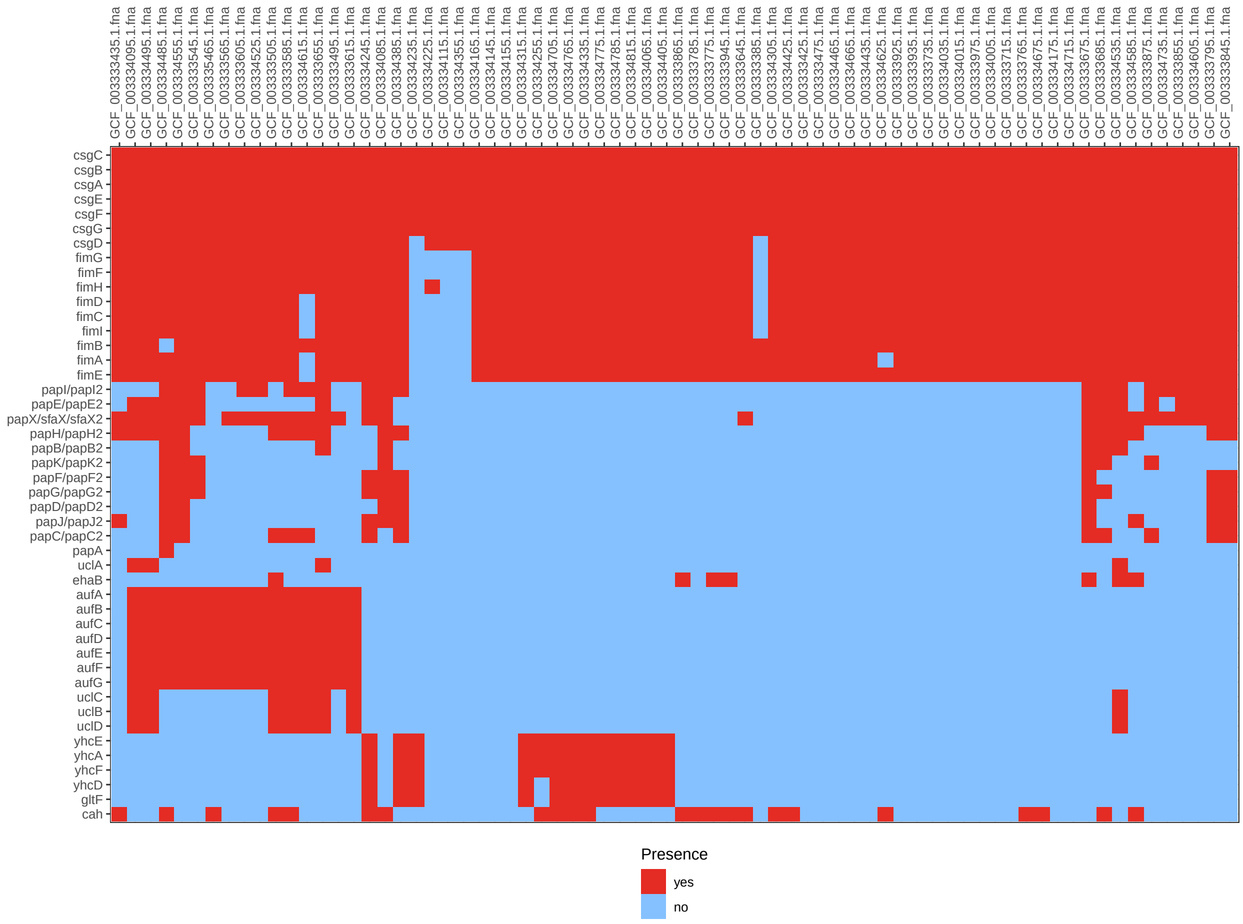

You can do the same for the number of copies:

```
get_presence_plot(presence_table = copies_df,
                  systems = c("Type_1", "Auf", "Yhc", "Curli",
                              "P_1", "UCL", "ehaB", "cah", "paa"))
```

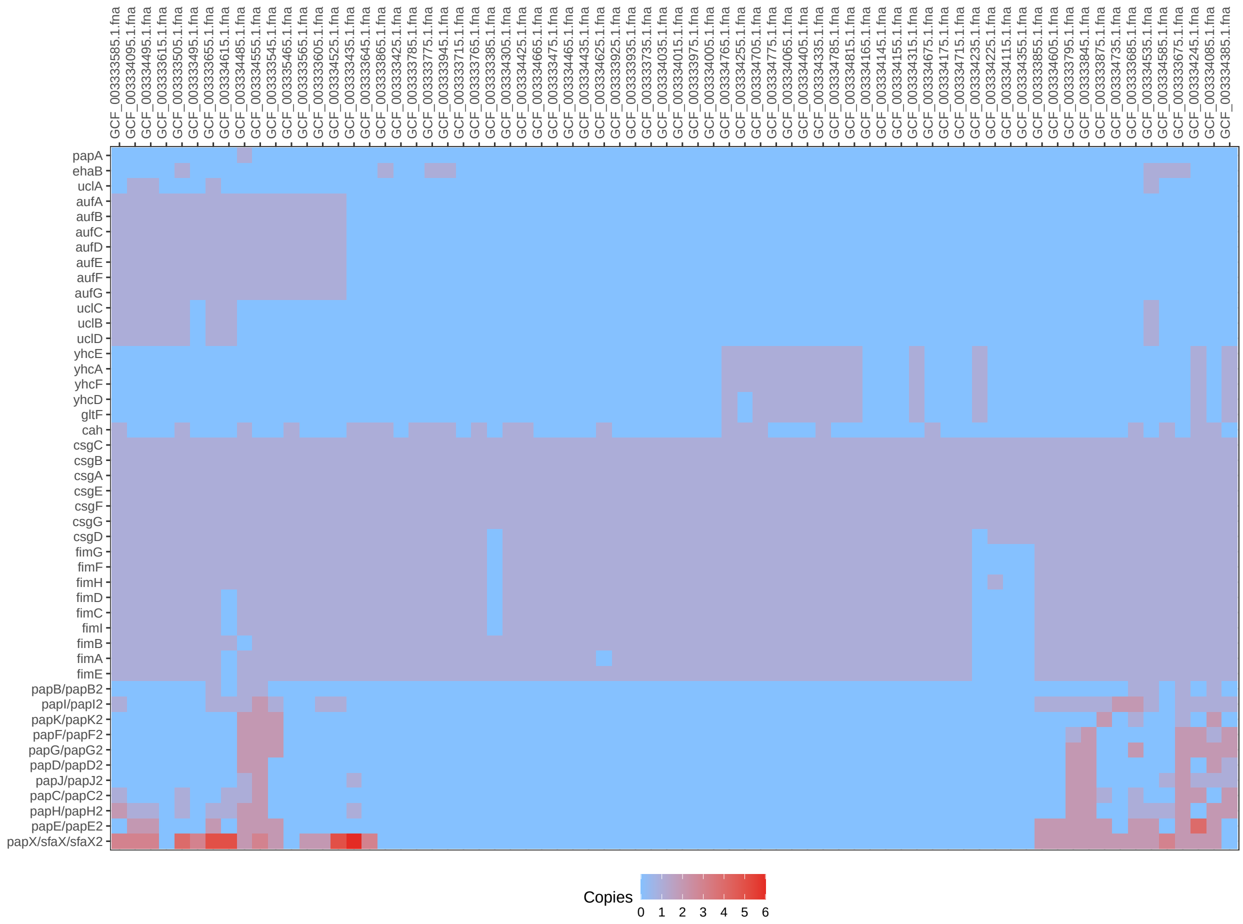

Analysis on system level

Get system information from gene presence. A system is considered as present if all of its genes are found.

```
system_df <- get_summary_table(presence_df)
```

You can save the results to a csv file:

```
write.csv(system_df, file = "Your_filename_systems.csv", row.names = FALSE)
```

Plotting results on system level

Again, you can plot all results:

```
get_summary_plot(presence_df)
```

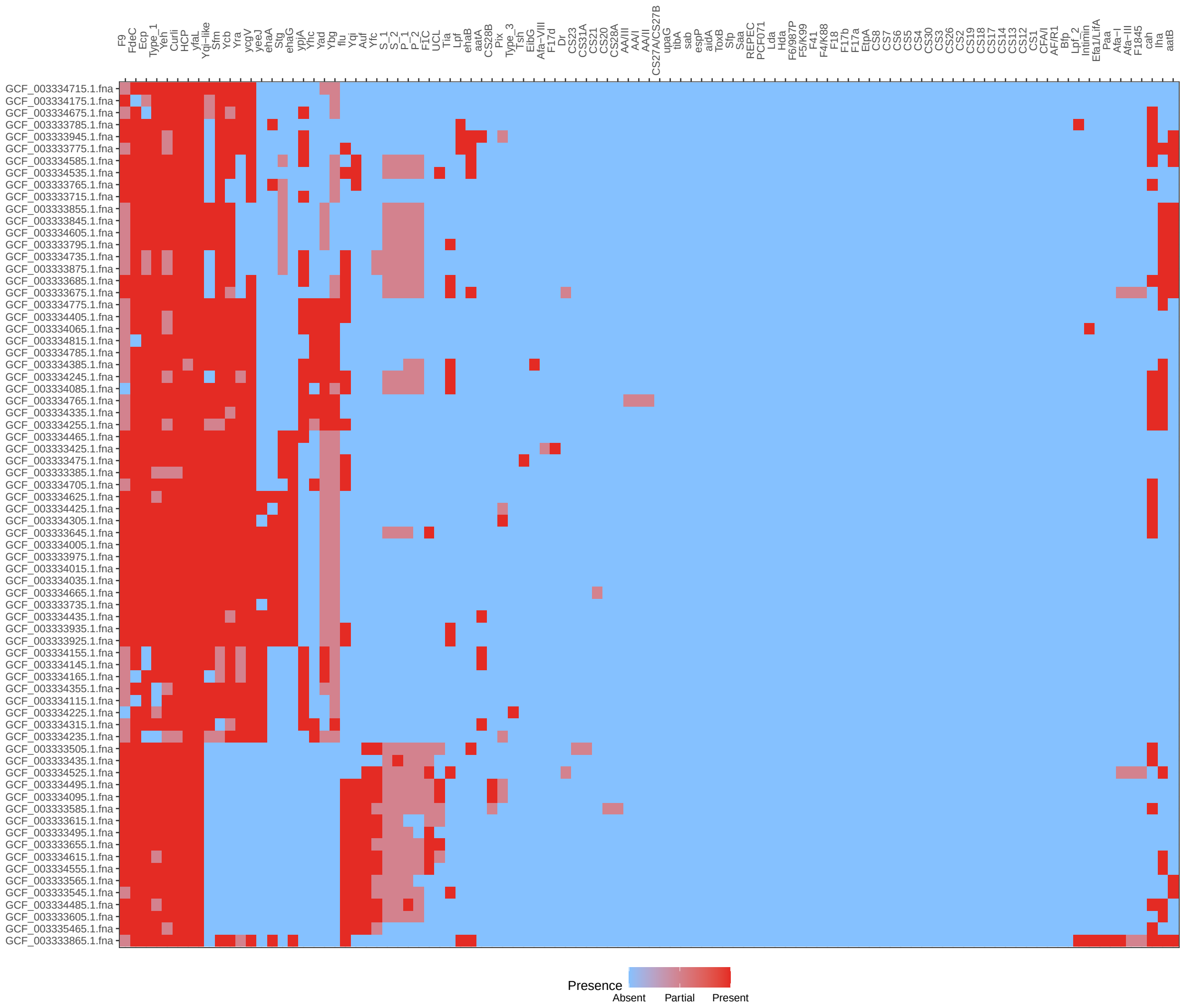

You can also skip systems that were not present in any of the analysed genomes using `hide_absent` argument:

```
get_summary_plot(presence_df,
                 hide_absent = TRUE)
```

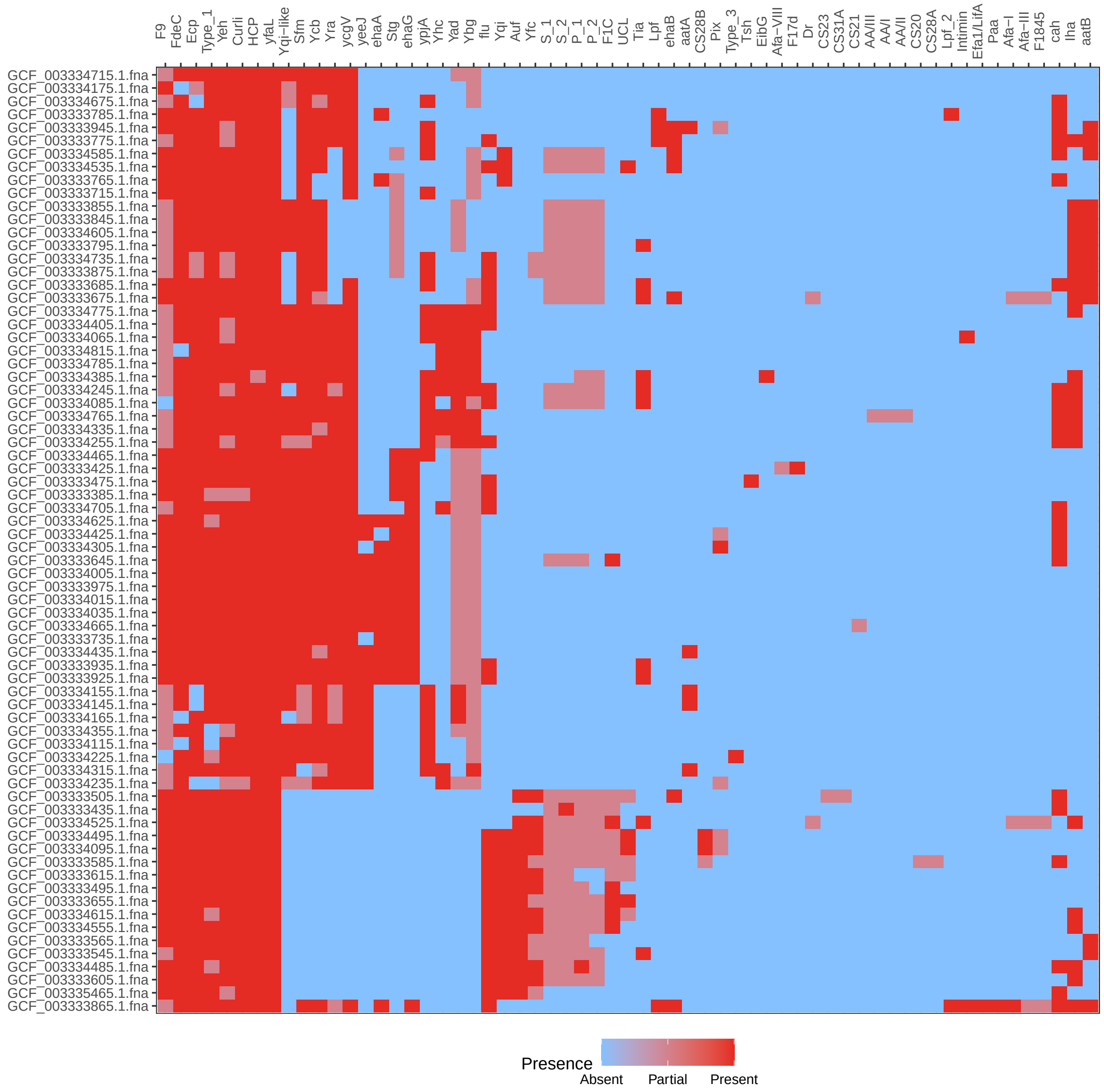

You can also modify plot colors by modifying `presence_col` and `absence_col` arguments:

```
get_summary_plot(presence_df,
                 hide_absent = TRUE,
                 presence_col = "#139e3d",
                 absence_col = "#bad1c1")
```

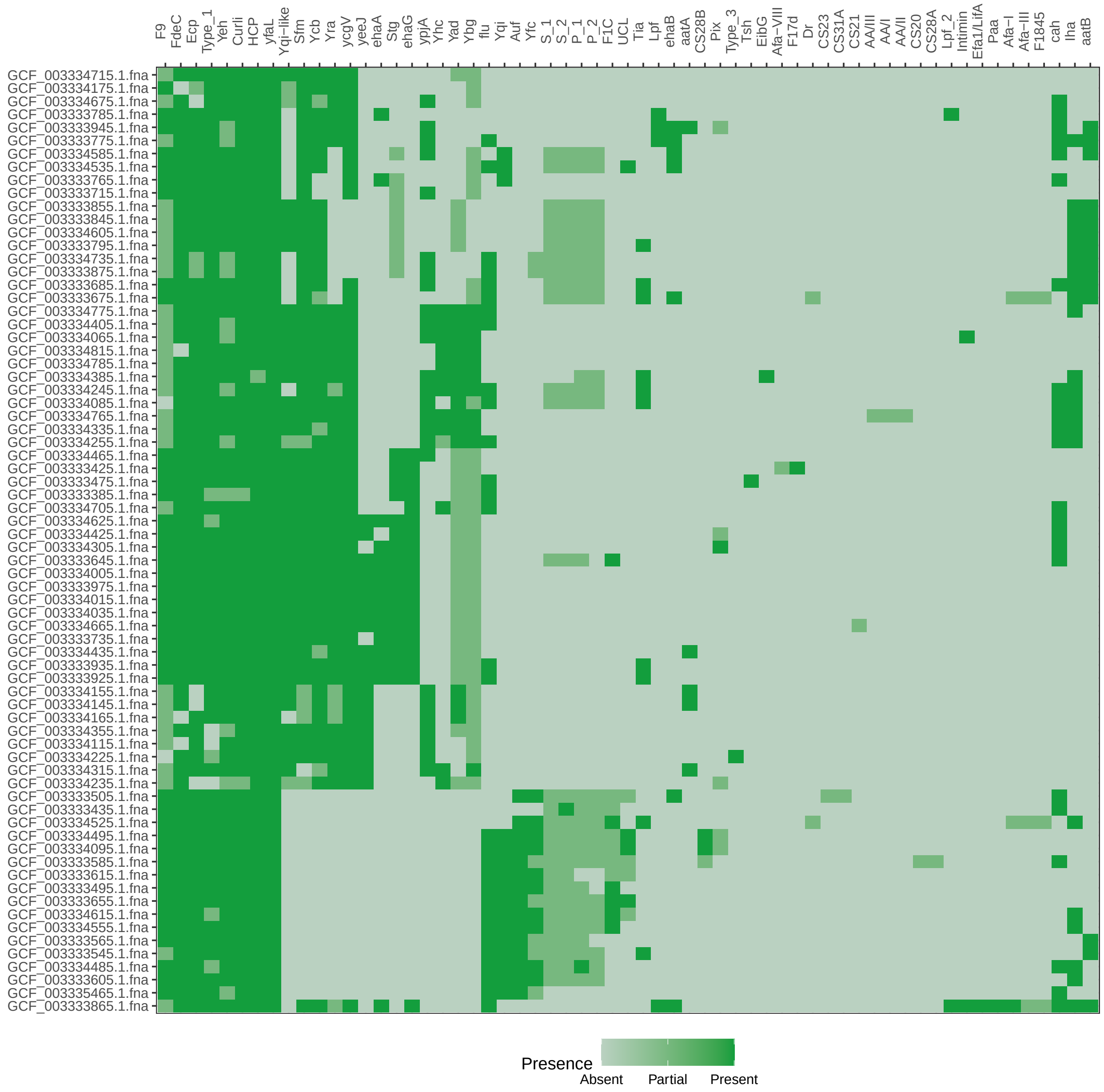

Profile analysis

Assign analysed genomes to adhesin profiles:

```r
profile_df <- get_adhesin_profiles(presence_df)
```

Let's take a look at results for a few genomes. If you notice `NA` in a profile assignment, it means that the analysed assembly has adhesin profile we did not identify in our genome collection.

```r
head(profile_df) %>%
  kbl()
```

File	Adhesins_profile	Fimbrial_profile	Nonfimbrial_profile
GCF_003333385.1.fna	A-4880	F-3224	N-30
GCF_003333425.1.fna	A-1473	F-216	N-14
GCF_003333435.1.fna	NA	NA	N-35
GCF_003333475.1.fna	A-3351	F-6	N-129
GCF_003333495.1.fna	A-230	F-87	N-18
GCF_003333505.1.fna	A-6611	F-4435	N-82

Cluster analysis

Assign analysed genomes to adhesin clusters:

```
cluster_df <- get_adhesin_clusters(presence_df)
```

Let's take a look at cluster assignments for a few genomes. Newly analysed assemblies are assigned to the cluster that matches their adhesin gene pattern most closely.

```
head(cluster_df) %>%
  kbl()
```

File	Adhesins_cluster	Fimbrial_cluster	Nonfimbrial_cluster
GCF_003333385.1.fna	A-A	F-C	N-D
GCF_003333425.1.fna	A-A	F-C	N-D
GCF_003333435.1.fna	A-I	F-G	N-D
GCF_003333475.1.fna	A-A	F-C	N-D
GCF_003333495.1.fna	A-F	F-B	N-D
GCF_003333505.1.fna	A-F	F-B	N-D

Report generation

With adhesiomeR you can also generate HTML report with results of all your analyses. It generates a directory with HTML report, all tables in csv format and all plots in png format. By default it will include all results but if you wish to save only specific results, you can do that by modifying `elements` argument. Allowed elements are: `presence_table`, `presence_plot`, `summary_table`, `summary_plot`, `profile_table`, `cluster_table`.

```
generate_report(presence_df,
                outdir = ".")
```

During generation of report, adhesiomeR creates intermediate files (tables in csv format and plots in png format). By default, these are not removed. If you wish to retain only the HTML report, please set `remove_intermediate_files` argument to `TRUE`.

```
generate_report(presence_df,
                outdir = ".",
                remove_intermediate_files = TRUE)
```

You can also define if plots in the report should display genes and systems that were not found in any of the assemblies by setting `hide_absent_genes` and `hide_absent_systems` arguments to `TRUE`.

```
generate_report(presence_df,
                outdir = ".",
                hide_absent_genes = TRUE,
                hide_absent_systems = TRUE)
```

Run adhesiomeR analysis - relaxed version

The relaxed version of the search is meant for more broad searches and identification of potentially novel adhesins. In this setting, you can set identity percent and coverage thresholds.

Analysis on gene level

We will use ECOR genomes as an example, you can download them by following instructions in the first section of the tutorial.

```
genomes <- list.files("./ecor_genomes/", full.names = TRUE)
```

Now, you can run blast search and specify number of threads to use with `n_threads` argument:

```
library(adhesiomeR)
blast_results <- get_blast_res(genomes, n_threads = 4)
```

BLAST analysis will create some temporary output files that are deleted after successful BLAST run. By default, they are placed in the current working directory but you can change it using `tmp_dir` argument. Note that it should be an existing directory to which you have write access.

```
blast_results <- get_blast_res(genomes, n_threads = 4, tmp_dir = "./tmp")
```

The next step is to get gene presence information from blast results. Here, you can set the thresholds for considering gene as present or absent. By default, adhesiomeR uses 75% thresholds for both. In the resulting table, 1 indicates gene presence and 0 its absence.

```
presence_rel <- get_presence_table_relaxed(blast_res = blast_results,
                                           n_threads = 8)
```

You can save the results to a csv file:

```
write.csv(presence_rel, file = "Your_filename.csv", row.names = FALSE)
```

You can modify the default thresholds using `identity` and `coverage` arguments:

```
presence_rel2 <- get_presence_table_relaxed(blast_res = blast_results,
                                            identity = 90,
                                            coverage = 90,
                                            n_threads = 8)
```

If you wish to see only genes that were found in at least one file, you can set `add_missing` argument to `FALSE`. Note that by default the results include all genes from the adhesiomeR database.

```r
presence_rel3 <- get_presence_table_relaxed(blast_res = blast_results,
                                            n_threads = 8,
                                            add_missing = FALSE)
```

If you are interested in number of gene copies, you can use `count_copies` argument:

```r
copies_rel <- get_presence_table_strict(blast_res = blast_results,
                                        n_threads = 8,
                                        count_copies = TRUE)
```

Plotting results on gene level

You can easily plot the presence/absence of adhesin genes. For simplicity (and due to the size of the full plot), we will plot only a few systems: type 1, Auf, Yhc, Pix, UCL fimbriae, ehaB, cah and paa. By default (without specifying `systems` argument), genes from all systems will be plotted. Note that if you analyse more than one genome, the results on a heatmap are clustered for more clear visualisation.

```r
get_presence_plot(presence_table = presence_rel,
                  systems = c("Type_1", "Auf", "Yhc", "Curli",
                              "P_1", "UCL", "ehaB", "cah", "paa"))
```

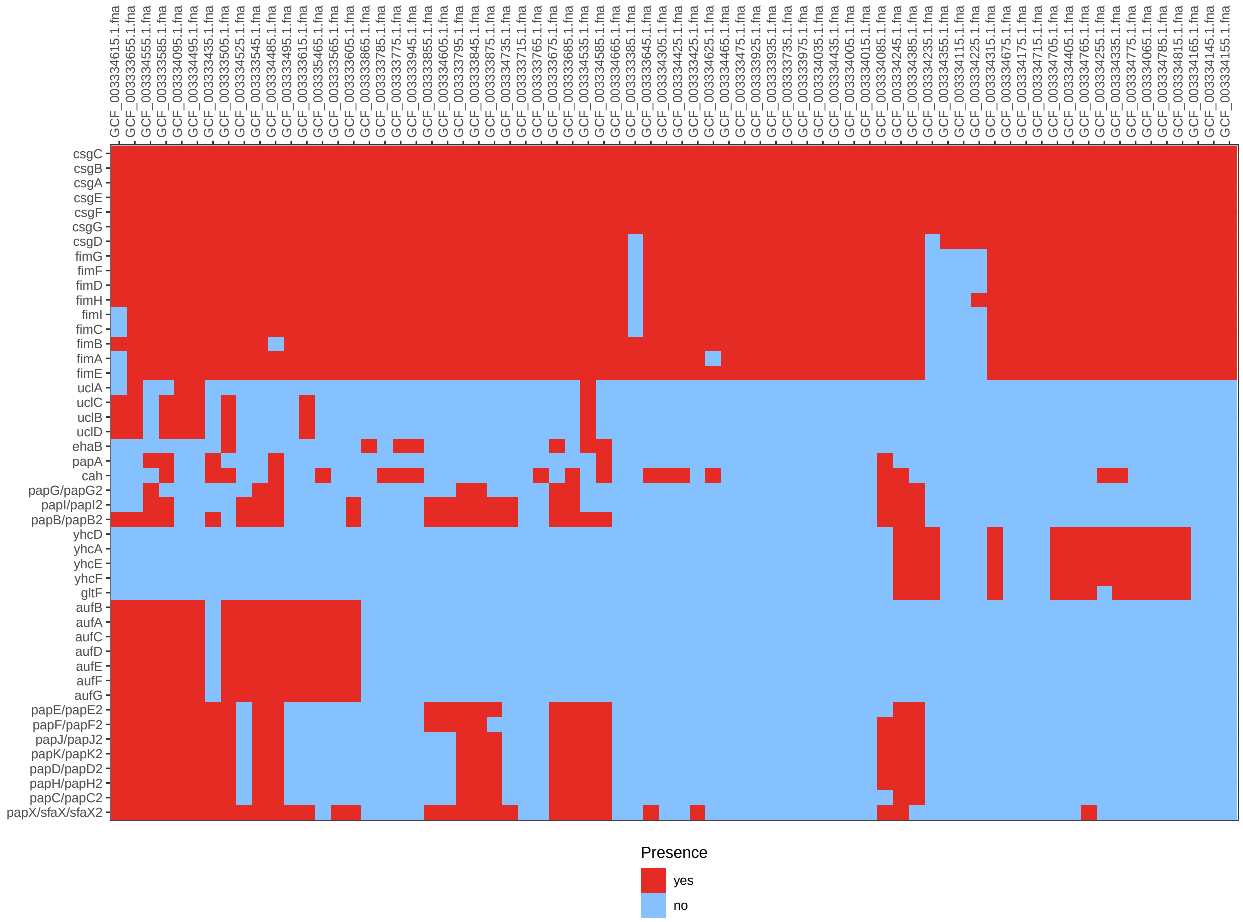

The plot above used results obtained with default thresholds (75% identity and coverage). Let's see how it looks like when we plot results obtained with thresholds set to 90%.

```r
get_presence_plot(presence_table = presence_rel2,
                  systems = c("Type_1", "Auf", "Yhc", "Curli",
                              "P_1", "UCL", "ehaB", "cah", "paa"))
```

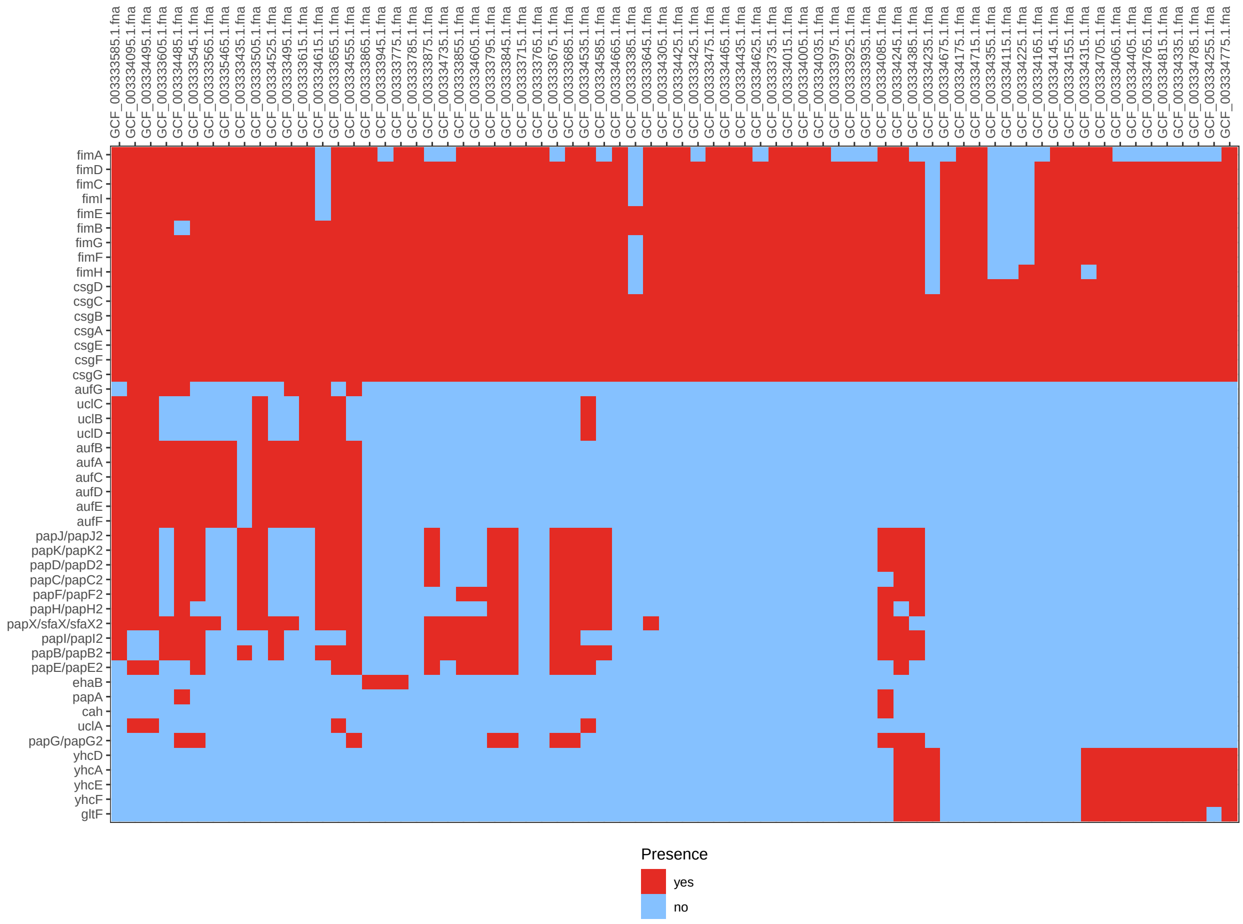

You can do the same plots for the number of copies:

```r
get_presence_plot(presence_table = copies_rel,
                  systems = c("Type_1", "Auf", "Yhc", "Curli",
                              "P_1", "UCL", "ehaB", "cah", "paa"))
```

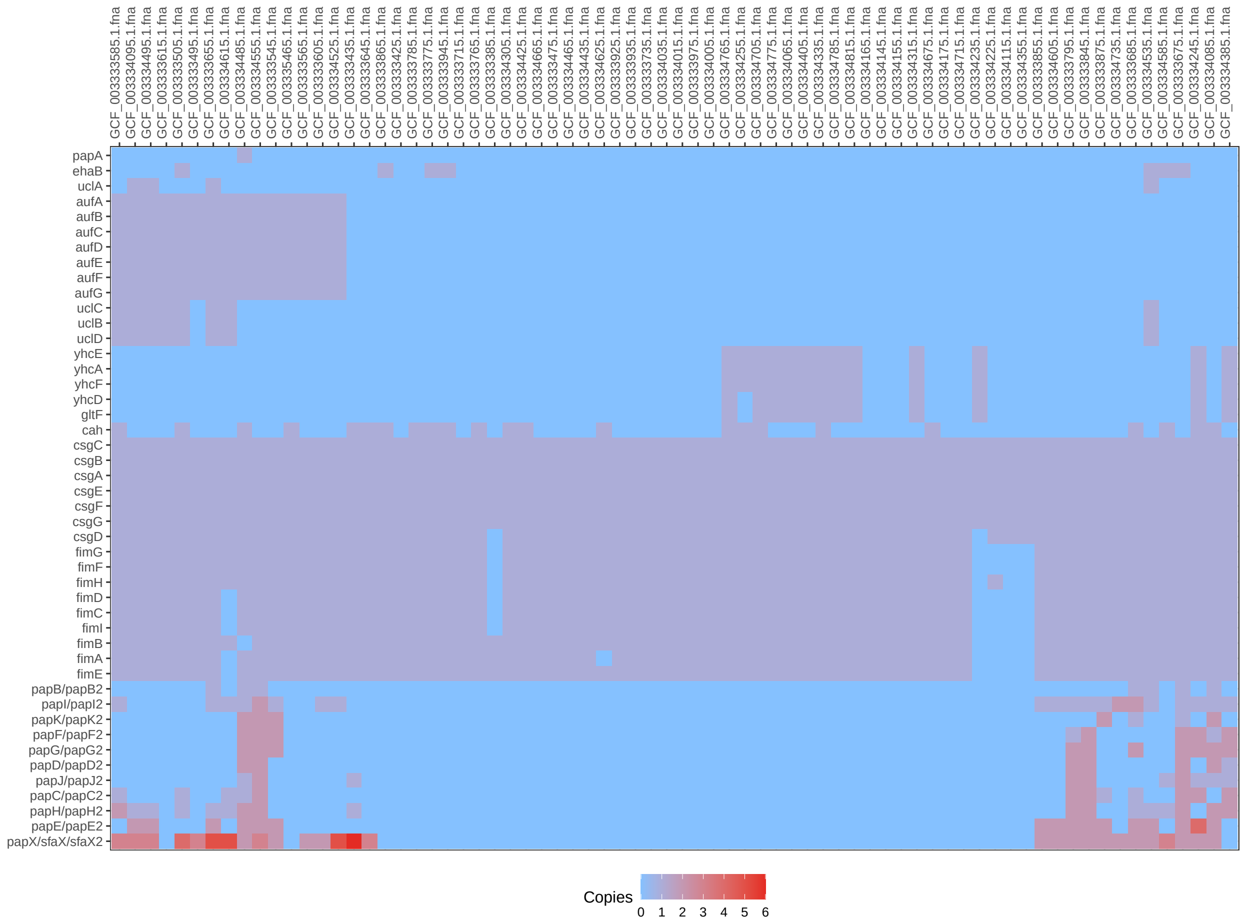

Analysis on system level

Get system information from gene presence. A system is considered as present if all of its genes are found.

```
system_rel <- get_summary_table(presence_rel)
```

You can save the results to a csv file:

```
write.csv(system_rel, file = "Your_filename_systems.csv", row.names = FALSE)
```

Plotting results on system level

Again, you can plot all results:

```
get_summary_plot(presence_rel)
```

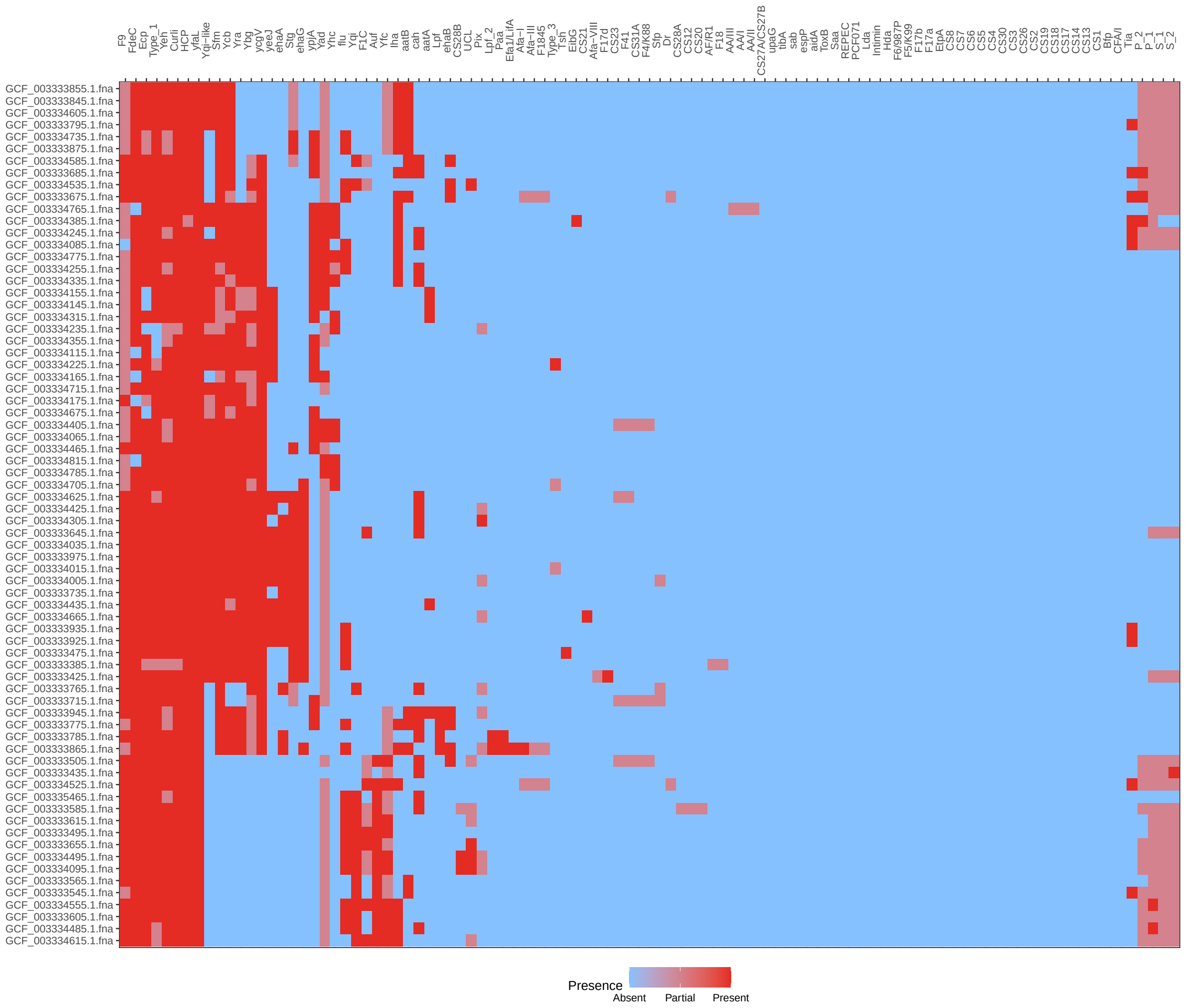

You can also skip systems that were not present in any of the analysed genomes using `hide_absent` argument:

```
get_summary_plot(presence_rel,
                 hide_absent = TRUE)
```

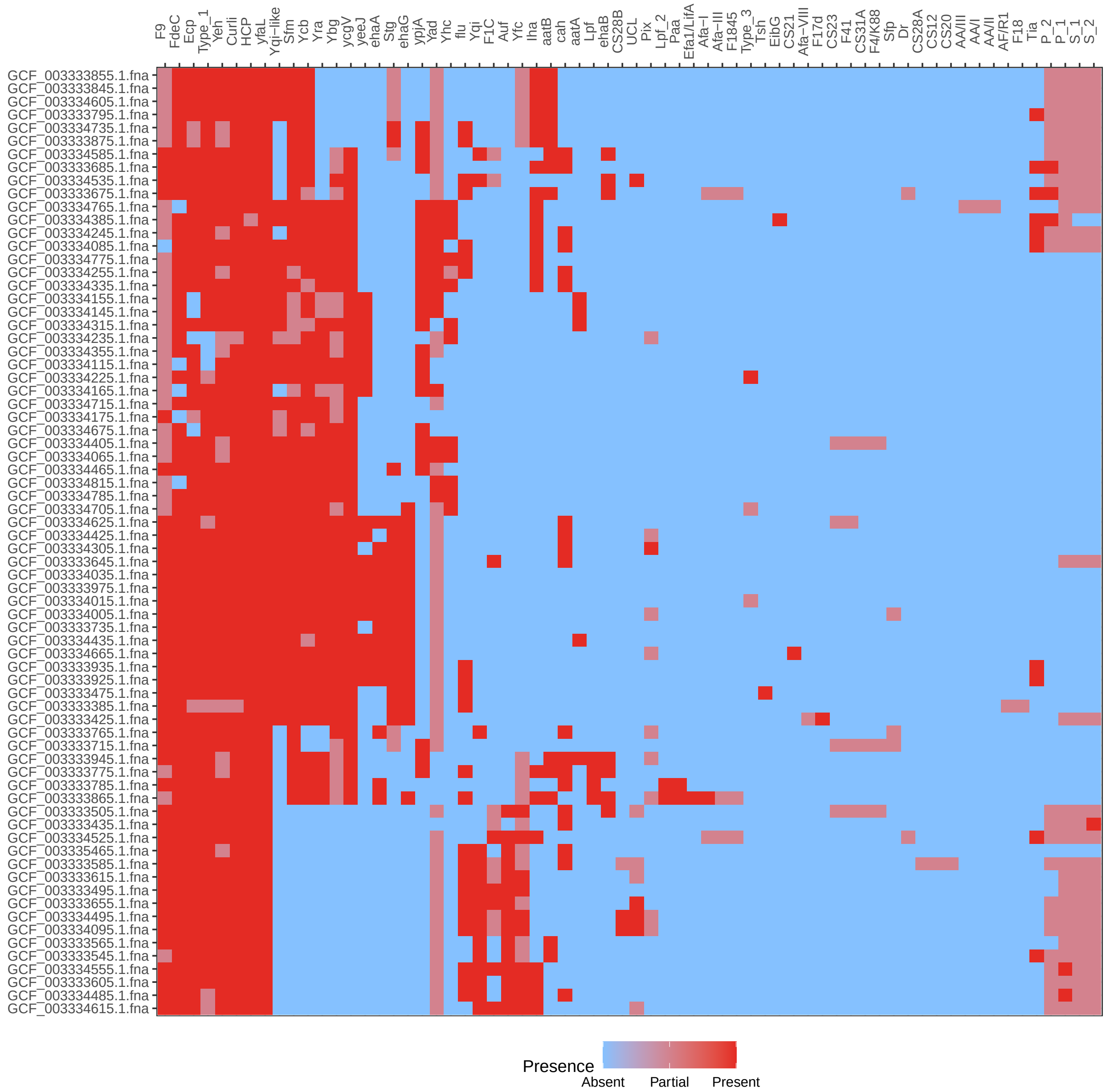

You can also modify plot colors by modifying `presence_col` and `absence_col` arguments:

```
get_summary_plot(presence_rel,
                 hide_absent = TRUE,
                 presence_col = "#139e3d",
                 absence_col = "#bad1c1")
```

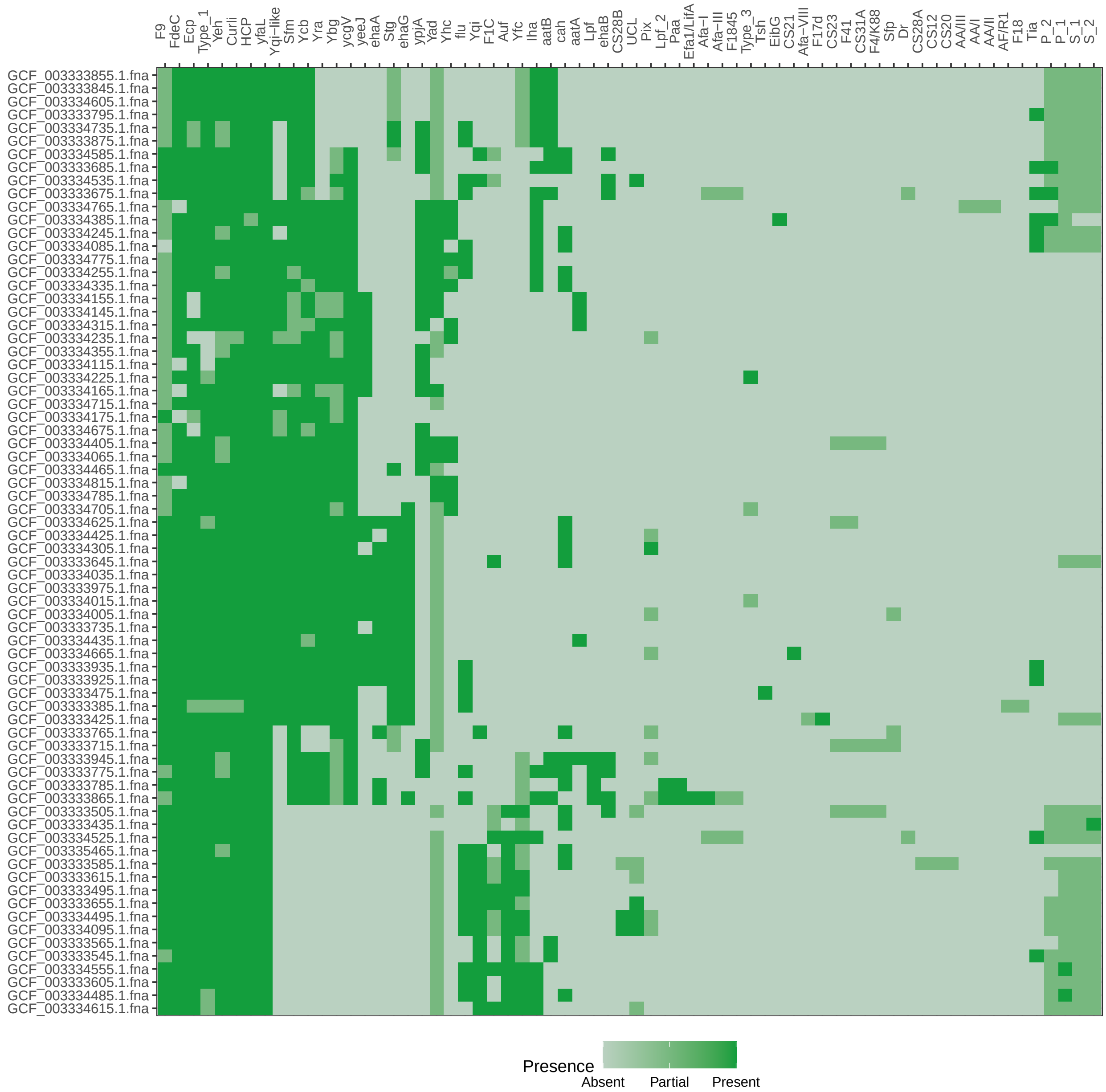

Report generation

With adhesiomeR you can also generate HTML report with results of all your analyses. It generates a directory with HTML report, all tables in csv format and all plots in png format. By default it will include all results but if you wish to save only specific results, you can do that by modifying `elements` argument.

```
generate_report(presence_rel,
                outdir = ".")
```

During generation of report, adhesiomeR creates intermediate files (tables in csv format and plots in png format). By default, these are not removed. If you wish to retain only the HTML report, please set `remove_intermediate_files` argument to `TRUE`.

```r
generate_report(presence_rel2,
                outdir = ".",
                remove_intermediate_files = TRUE)
```

You can also define if plots in the report should display genes and systems that were not found in any of the assemblies by setting `hide_absent_genes` and `hide_absent_systems` arguments to `TRUE`.

```r
generate_report(presence_rel3,
                outdir = ".",
                hide_absent_genes = TRUE,
                hide_absent_systems = TRUE)
```
