# Supplementary material for: adhesiomeR: a tool for Escherichia coli adhesin classification and analysis
Source: BMC Genomics. 2024 Jun 17;25:609. doi: 10.1186/s12864-024-10525-6 (PMC11184843; doi:10.1186/s12864-024-10525-6)
Supplement: Supplementary file 9 — Additional file 9: Tutorial_pangenomes. Tutorial describing how to run adhesiomeR analysis of a pangenome using R package. [file 12864_2024_10525_MOESM9_ESM.pdf]

Analysis of pangenomes with adhesiomeR

Contents

Example files . . . 1
Run adhesiomeR analysis - strict version . . . 1
Analysis on gene level . . . 1
Plotting results on gene level . . . 2
Analysis on system level . . . 4
Plotting results on system level . . . 4
Run adhesiomeR analysis - relaxed version . . . 7
Analysis on gene level . . . 7
Plotting results on gene level . . . 8
Analysis on system level . . . 10
Plotting results on system level . . . 10

Example files

This step is optional. If you already have your own genome pangenomes you would like to analyse with adhesiomeR, proceed to the next step. Here, we will be using pangenome built with panaroo using 72 ECOR strains. You can download example files below:

- pan_genome_reference.fa
- gene_presence_absence.csv

Run adhesiomeR analysis - strict version

This version of adhesiomeR search is meant for identification of known adhesins.

Analysis on gene level

First, you need to run blast search on the pangenome. You can specify number of threads to use with `n_threads` argument:

```
library(adhesiomeR)
blast_results <- get_blast_res("pan_genome_reference.fa", n_threads = 8)
```

The next step is to extend the results obtained for the pangenome to individual genomes based on the gene presence/absence matrix:

```
blast_results_full <- pangenome_to_genome(blast_results, "gene_presence_absence.csv")
```

Now, we can use the extended results to obtain adhesin gene presence:

```
presence_df <- get_presence_table_strict(blast_res = blast_results_full,
                                         n_threads = 8)
```

If you wish to see only genes that were found in at least one file, you can set `add_missing` argument to `FALSE`. Note that by default the results include all genes from the adhesiomeR database.

```
presence_df2 <- get_presence_table_strict(blast_res = blast_results_full,
                                          n_threads = 8,
                                          add_missing = FALSE)
```

Please note that due to the nature of the pangenome, it is not possible to determine adhesin gene copy number using this approach. It is also not recommended to use profile and cluster assignment as they have been developed to be used mainly with individual genome assemblies.

Plotting results on gene level

You can easily plot the presence/absence of adhesin genes. For simplicity (and due to the size of the full plot), we will plot only a few systems: type 1, Auf, Yhc, Pix, UCL fimbriae, ehaB, cah and paa. By default (without specifying `systems` argument), genes from all systems will be plotted. Note that if you analyse more than one genome, the results on a heatmap are clustered for more clear visualisation.

```
get_presence_plot(presence_table = presence_df,
                  systems = c("Type_1", "Auf", "Yhc", "Curli",
                               "P_1", "UCL", "ehaB", "cah", "paa"))
```

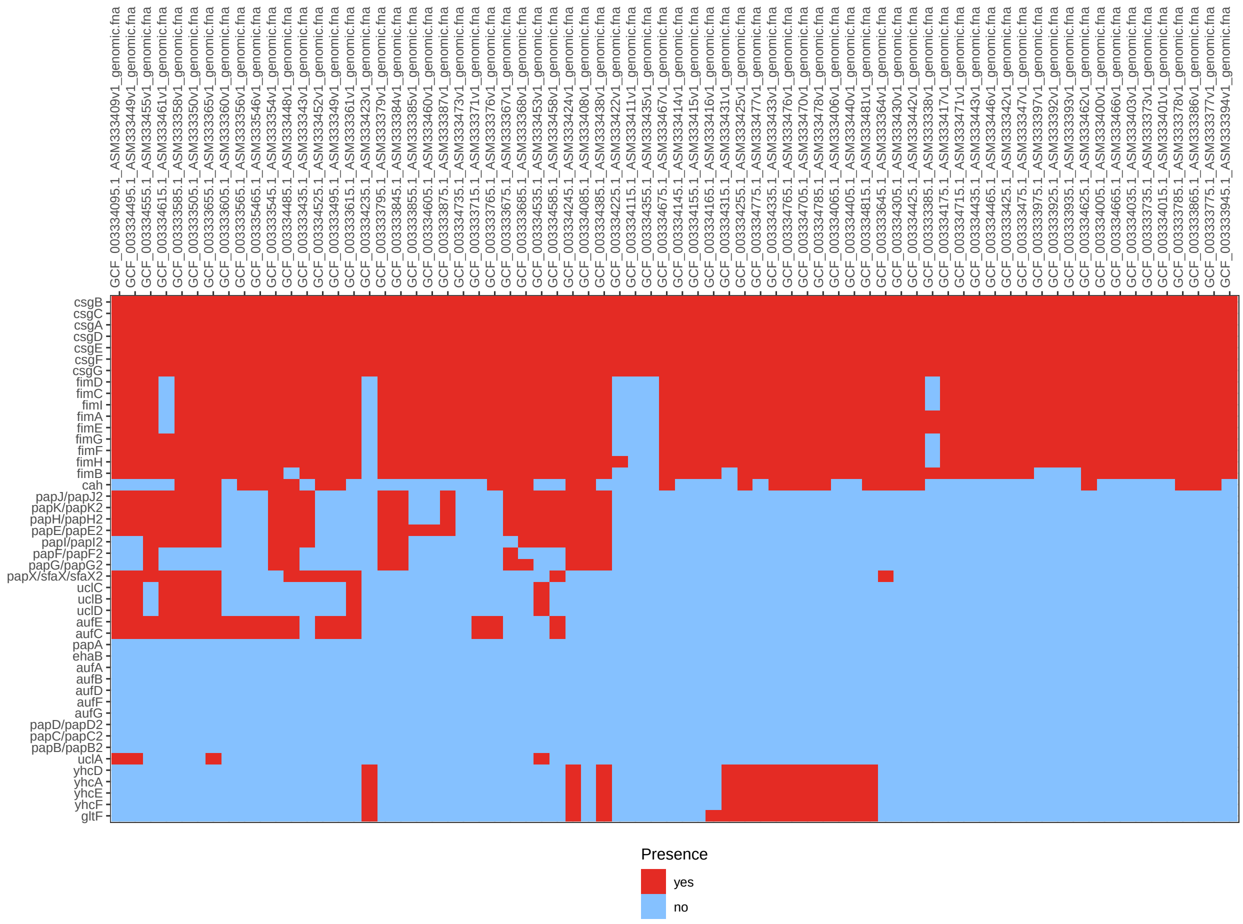

You can do the same for the number of copies:

```r
get_presence_plot(presence_table = copies_df,
                  systems = c("Type_1", "Auf", "Yhc", "Curli",
                              "P_1", "UCL", "ehaB", "cah", "paa"))
```

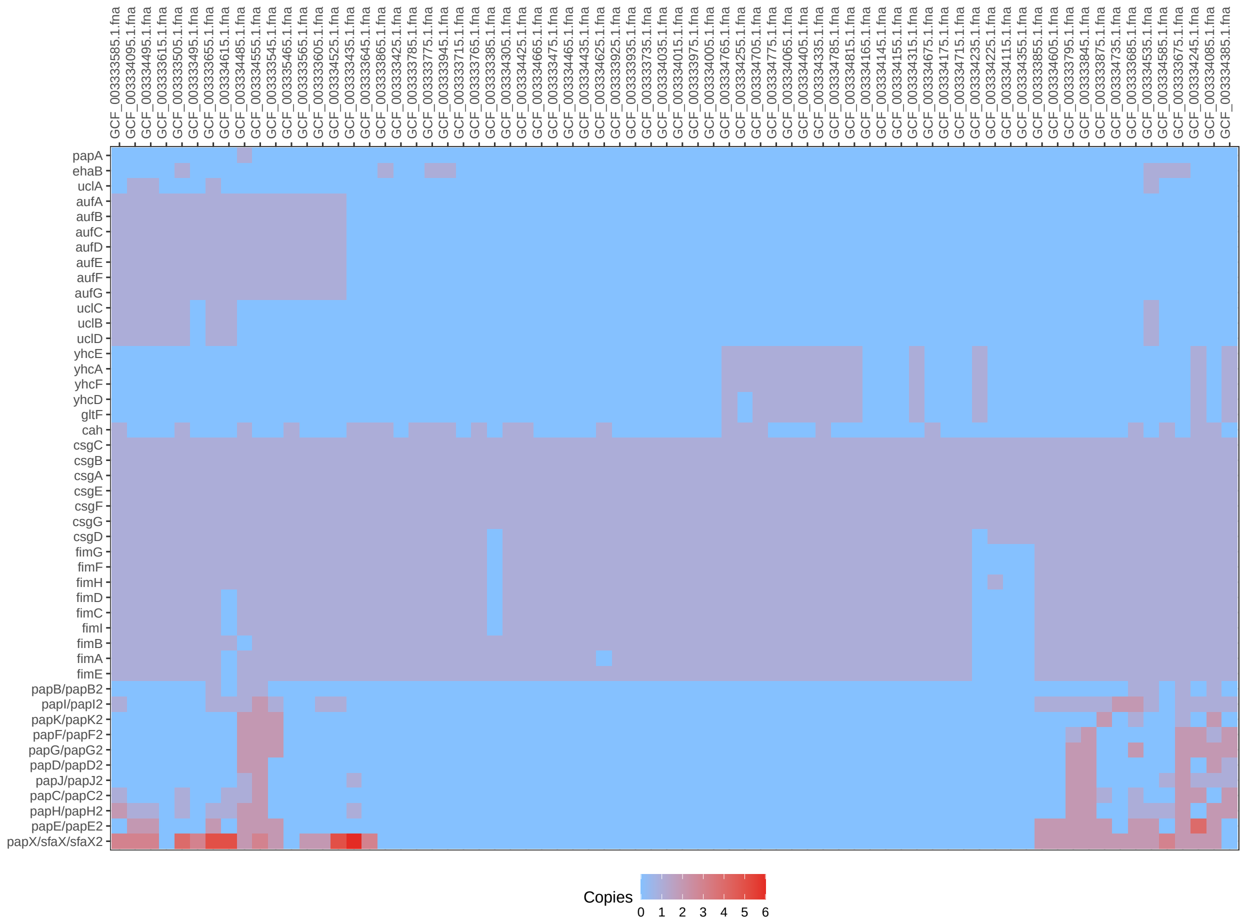

Analysis on system level

Get system information from gene presence. A system is considered as present if all of its genes are found.

```
system_df <- get_summary_table(presence_df)
```

Plotting results on system level

Again, you can plot all results:

```
get_summary_plot(presence_df)
```

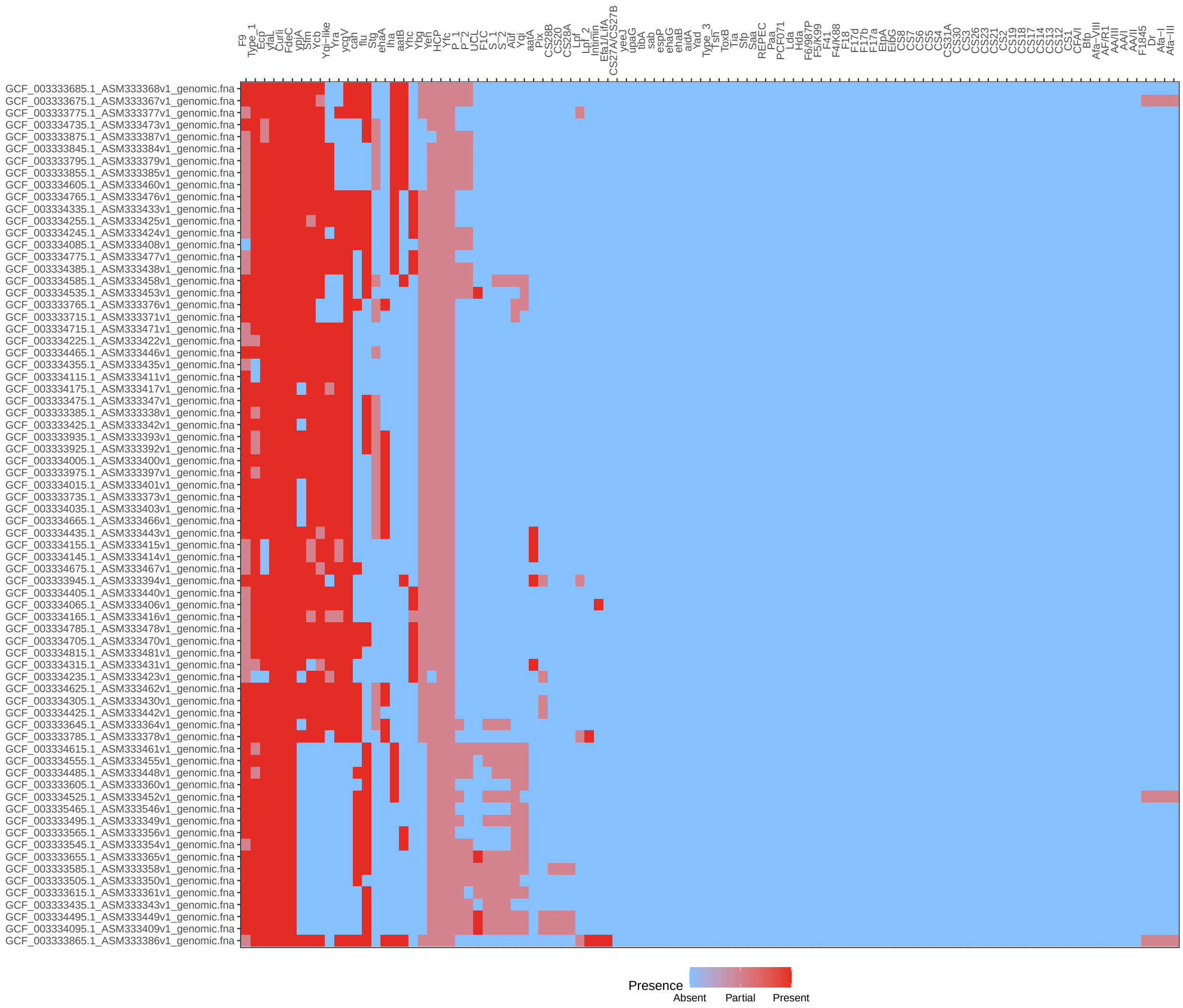

You can also skip systems that were not present in any of the analysed genomes using `hide_absent` argument:

```
get_summary_plot(presence_df,
                 hide_absent = TRUE)
```

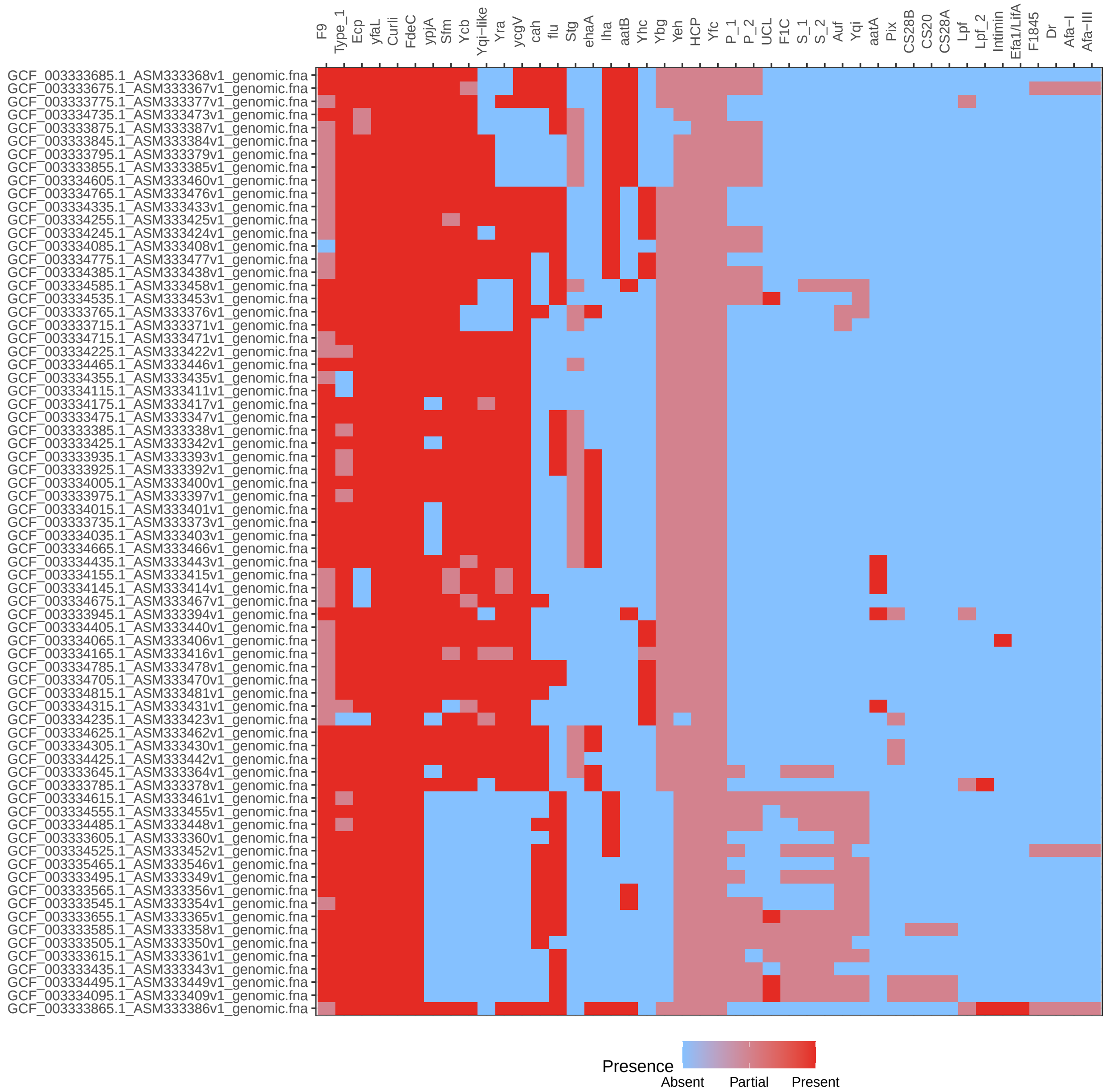

You can also modify plot colors by modifying `presence_col` and `absence_col` arguments:

```
get_summary_plot(presence_df,
                 hide_absent = TRUE,
                 presence_col = "#139e3d",
                 absence_col = "#bad1c1")
```

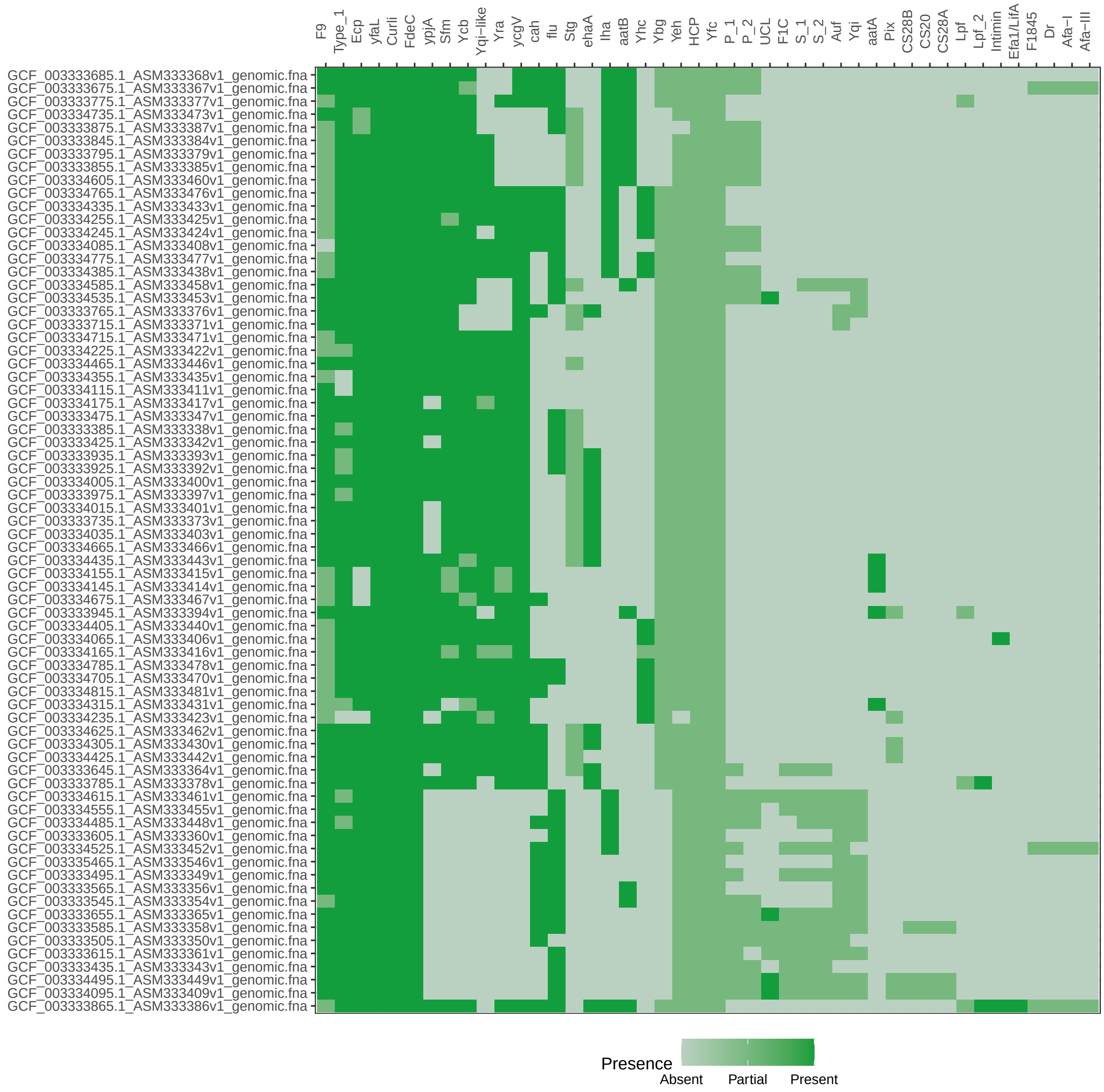

Run adhesiomeR analysis - relaxed version

The relaxed version of the search is meant for more broad searches and identification of potentially novel adhesins. In this setting, you can set identity percent and coverage thresholds.

We will use ECOR pangenome as an example, you can download example files by following instructions in the first section of the tutorial.

Analysis on gene level

First, you need to run blast search on the pangenome. You can specify number of threads to using `n_threads` argument:

```r
library(adhesiomeR)
blast_results <- get_blast_res("pan_genome_reference.fa", n_threads = 8)
```

The next step is to extend the results obtained for the pangenome to individual genomes based on the gene presence/absence matrix:

```r
blast_results_full <- pangenome_to_genome(blast_results, "gene_presence_absence.csv")
```

The next step is to get gene presence information from extended blast results. Here, you can set the thresholds for considering gene as present or absent. By default, adhesiomeR uses 75% thresholds for both. In the resulting table, 1 indicates gene presence and 0 its absence.

```r
presence_rel <- get_presence_table_relaxed(blast_res = blast_results_full,
                                           n_threads = 8)
```

You can modify the default thresholds using `identity` and `coverage` arguments:

```r
presence_rel2 <- get_presence_table_relaxed(blast_res = blast_results_full,
                                            identity = 90,
                                            coverage = 90,
                                            n_threads = 8)
```

If you wish to see only genes that were found in at least one file, you can set `add_missing` argument to `FALSE`. Note that by default the results include all genes from the adhesiomeR database.

```r
presence_rel3 <- get_presence_table_relaxed(blast_res = blast_results_full,
                                            n_threads = 8,
                                            add_missing = FALSE)
```

If you are interested in number of gene copies, you can use `count_copies` argument:

```r
copies_rel <- get_presence_table_strict(blast_res = blast_results_full,
                                        n_threads = 8,
                                        count_copies = TRUE)
```

Plotting results on gene level

You can easily plot the presence/absence of adhesin genes. For simplicity (and due to the size of the full plot), we will plot only a few systems: type 1, Auf, Yhc, Pix, UCL fimbriae, ehaB, cah and paa. By default (without specifying `systems` argument), genes from all systems will be plotted. Note that if you analyse more than one genome, the results on a heatmap are clustered for more clear visualisation.

```r
get_presence_plot(presence_table = presence_rel,
                  systems = c("Type_1", "Auf", "Yhc", "Curli",
                              "P_1", "UCL", "ehaB", "cah", "paa"))
```

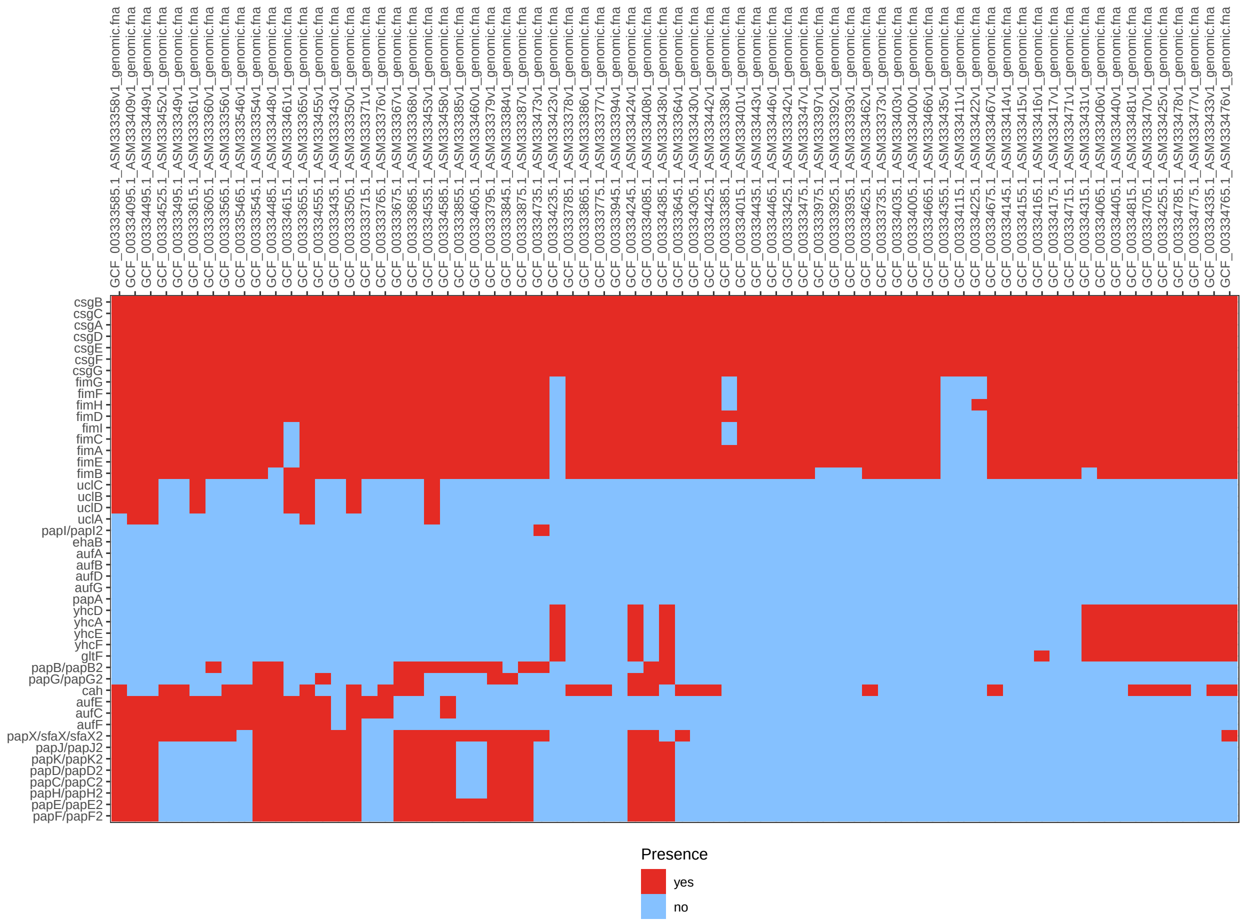

The plot above used results obtained with default thresholds (75% identity and coverage). Let's see how it looks like when we plot results obtained with thresholds set to 90%.

```
get_presence_plot(presence_table = presence_rel2,
                  systems = c("Type_1", "Auf", "Yhc", "Curli",
                              "P_1", "UCL", "ehaB", "cah", "paa"))
```

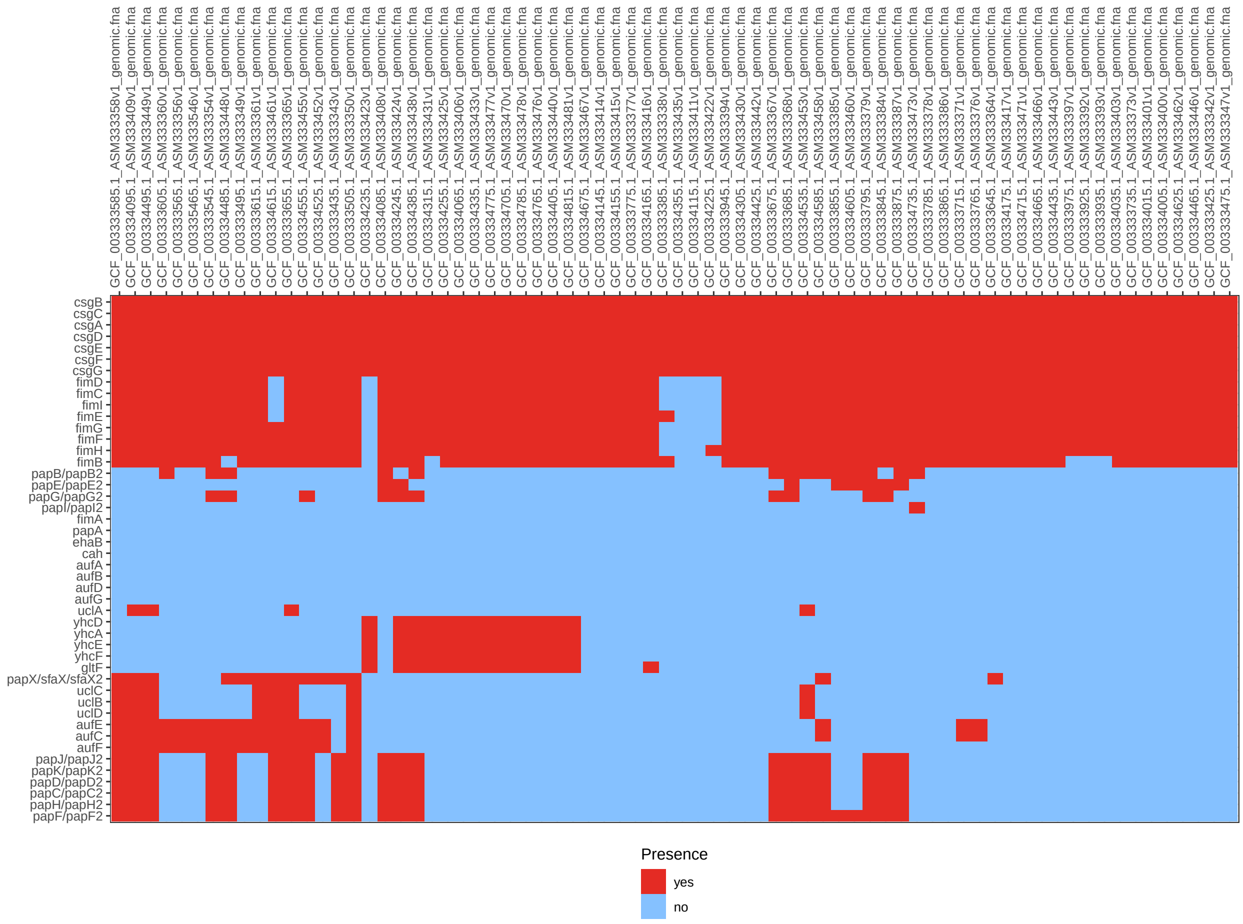

Analysis on system level

Get system information from gene presence. A system is considered as present if all of its genes are found.

```
system_rel <- get_summary_table(presence_rel)
```

Plotting results on system level

Again, you can plot all results:

```
get_summary_plot(presence_rel)
```

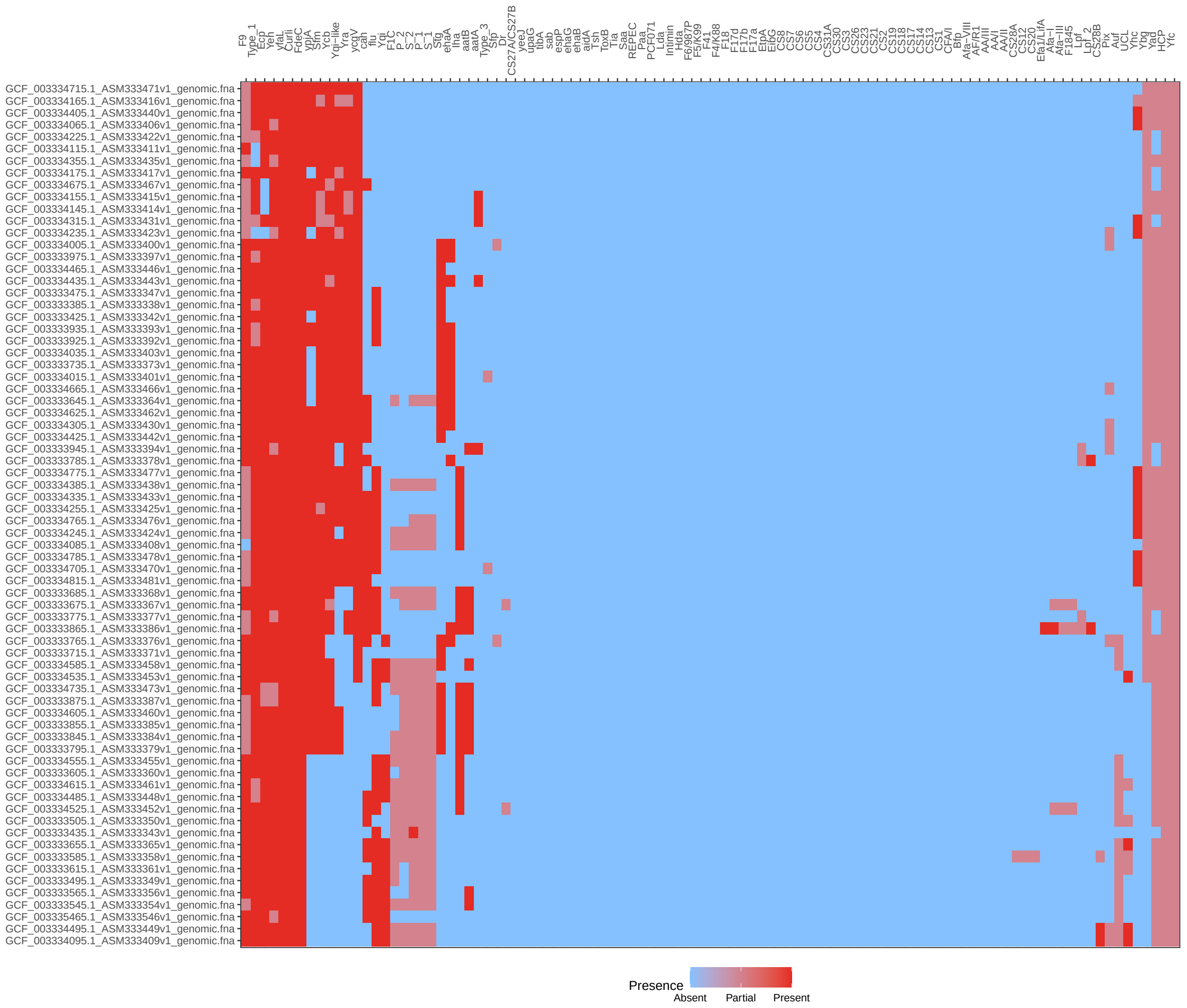

You can also skip systems that were not present in any of the analysed genomes using `hide_absent` argument:

```
get_summary_plot(presence_rel,
                 hide_absent = TRUE)
```

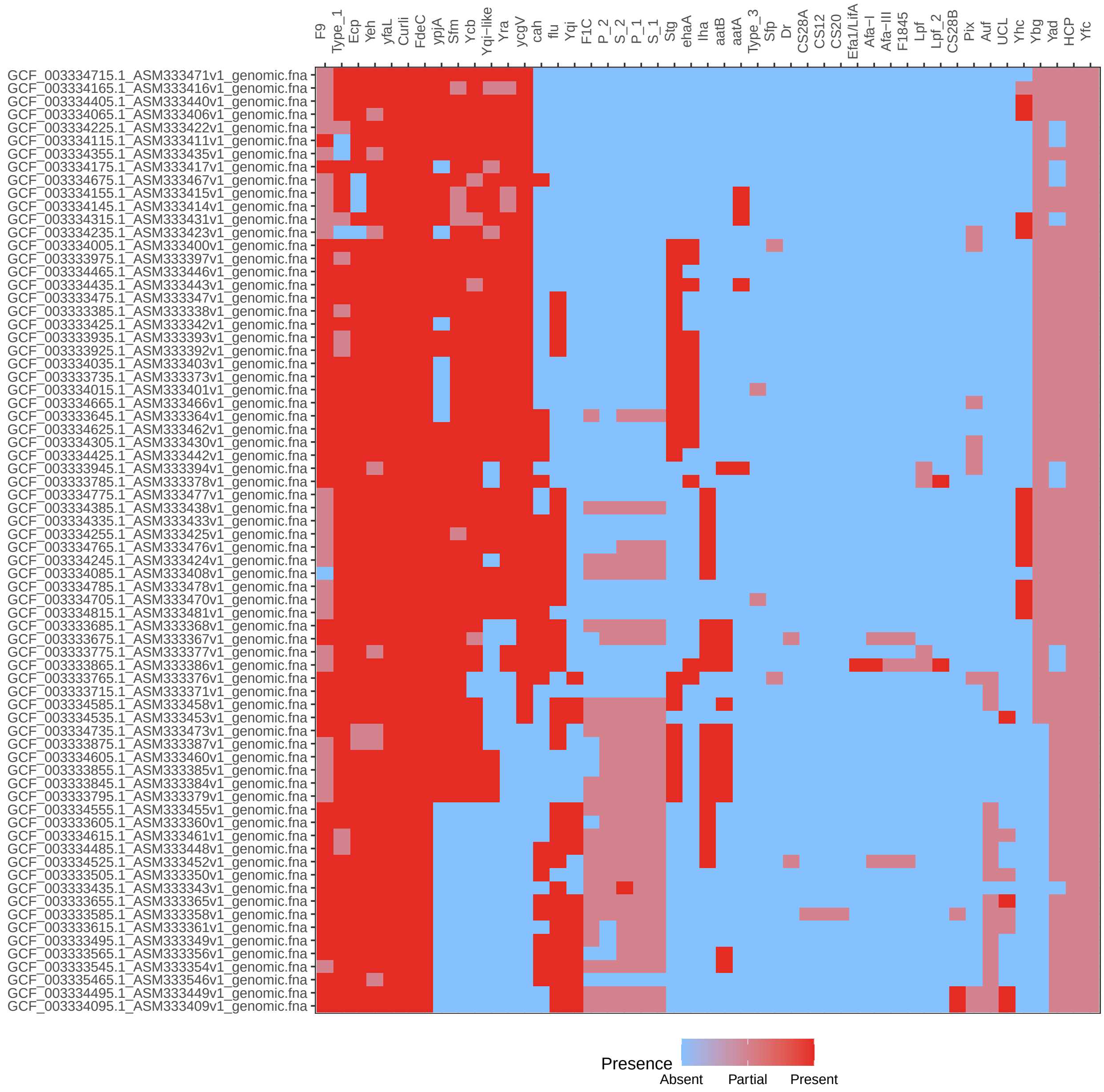

You can also modify plot colors by modifying `presence_col` and `absence_col` arguments:

```
get_summary_plot(presence_rel,
                 hide_absent = TRUE,
                 presence_col = "#139e3d",
                 absence_col = "#bad1c1")
```

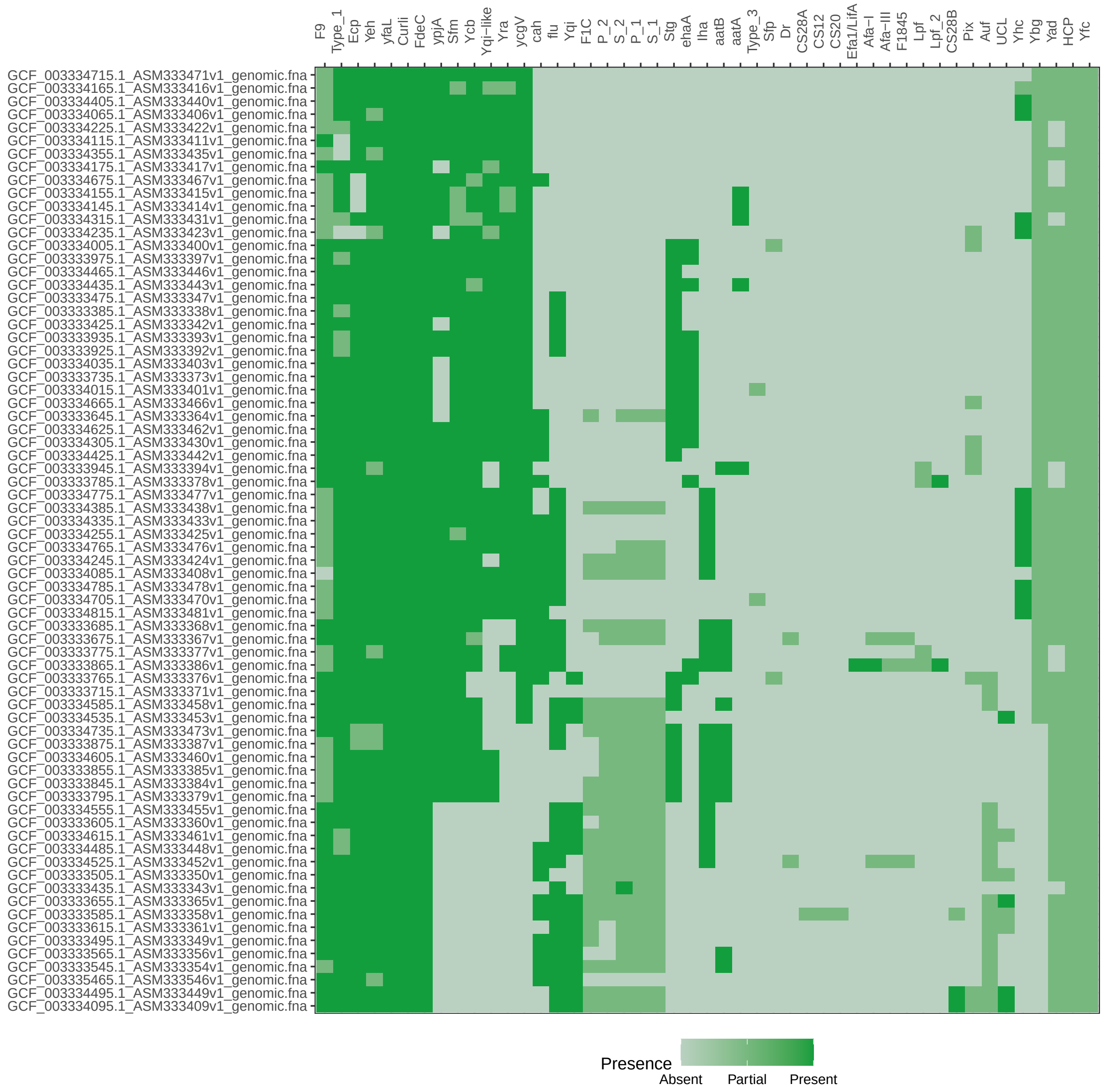

Please note that due to the nature of the pangenome, it is not possible to determine adhesin gene copy number using this approach. It is also not recommended to use profile and cluster assignment as they have been developed to be used mainly with individual genome assemblies.
