# Supplementary material for: adhesiomeR: a tool for Escherichia coli adhesin classification and analysis
Source: BMC Genomics. 2024 Jun 17;25:609. doi: 10.1186/s12864-024-10525-6 (PMC11184843; doi:10.1186/s12864-024-10525-6)
Supplement: Supplementary file 10 — Additional file 10: Tutorial_metagenomics. Tutorial describing how to run adhesiomeR analysis of metagenomic gene catalogue using R package. [file 12864_2024_10525_MOESM10_ESM.pdf]

Analysis of metagenomic data with adhesiomeR

Contents

Example files . . . 1
Run adhesiomeR analysis - strict version . . . 1
Analysis on gene level . . . 1
Plotting results on gene level . . . 2
Analysis on system level . . . 3
Plotting results on system level . . . 3
Run adhesiomeR analysis - relaxed version . . . 7
Analysis on gene level . . . 7
Plotting results on gene level . . . 8
Analysis on system level . . . 9
Plotting results on system level . . . 9

Example files

This example uses files published in Hildebrand et al. 2021. Please be aware that the following type of analysis is meant to be run on linux machine. First, we run BLAST search on a gene catalogue. In the next step, we want to associate each hit with samples that contain given gene. Since abundance matrices generated from metagenomic WGS analyses are generally too big to load them into R, we first extract the subset of data from the original file by selecting accessions/numbers of genes with hits to adhesins. This subset of the original abundance matrix can be loaded into R and used for further processing. Please note that this approach will not be able to identify multiple copies of adhesin genes in samples.

Run adhesiomeR analysis - strict version

This version of adhesiomeR search is meant for identification of known adhesins.

Analysis on gene level

First, you need to run blast search on the gene catalogue. You can specify number of threads to use with `n_threads` argument:

```r
library(adhesiomeR)
blast_results <- get_blast_res("compl.incompl.95.fna1", n_threads = 12)
```

Save blast results to csv file for further processing

```r
write.csv(blast_results, "gc_blast_results.csv", row.names = FALSE)
```

Extract subset of data from abundance matrix. Notice that this step does not use R but bash:

```bash
# Extract gene names with hits to adhesins
cut -d$'\t' -f 1 gc_blast_results.csv | uniq | grep -v "Query" > genes.txt

# Extract gene abundance in samples
zcat Matrix.mat.gz | head -n 1 > gene_abundance.txt
zcat Matrix.mat.gz | sed -nf <(sed 's/$/p/' genes.txt) >> gene_abundance.txt
```

You can also use these commands from within R using system function.

```
# Extract gene names with hits to adhesins
system("cut -d$'\t' -f 1 gc_blast_results.csv | uniq | grep -v 'Query' > genes.txt")

# Extract gene abundance in samples
system("zcat Matrix.mat.gz | head -n 1 > gene_abundance.txt")
system("zcat Matrix.mat.gz | sed -nf <(sed 's/$/p/' genes.txt) >> gene_abundance.txt")
```

The next step is to trace back the results obtained for the gene catalogue to individual samples based on abundance matrix:

```
# Define path to the subset of abundance matrix
ab_mat <- "gene_abundance.txt"

# Extend blast results
extended_blast_res <- gc_to_sample(blast_results, ab_mat)
```

Now, we can use the these results to obtain adhesin gene presence:

```
presence_df <- get_presence_table_strict(blast_res = extended_blast_res,
                                         n_threads = 8)
```

If you wish to see only genes that were found in at least one file, you can set `add_missing` argument to `FALSE`. Note that by default the results include all genes from the adhesiomeR database.

```
presence_df2 <- get_presence_table_strict(blast_res = extended_blast_res,
                                          n_threads = 8,
                                          add_missing = FALSE)
```

Please note that due to the nature of the pangenome, it is not possible to determine adhesin gene copy number using this approach. It is also not recommended to use profile and cluster assignment as they have been developed to be used mainly with individual genome assemblies.

Plotting results on gene level

You can easily plot the presence/absence of adhesin genes. For simplicity (and due to the size of the full plot), we will plot only a few systems: type 1, Auf, Yhc, Pix, UCL fimbriae, ehaB, cah and paa. By default (without specifying `systems` argument), genes from all systems will be plotted. Note that if you analyse more than one genome, the results on a heatmap are clustered for more clear visualisation. Due to the size of the gene catalogue, we will use only first 100 rows of data for this plot.

```
get_presence_plot(presence_table = presence_df[1:100, ],
                  systems = c("Type_1", "Auf", "Yhc", "Curli",
                              "P_1", "UCL", "ehaB", "cah", "paa"))
```

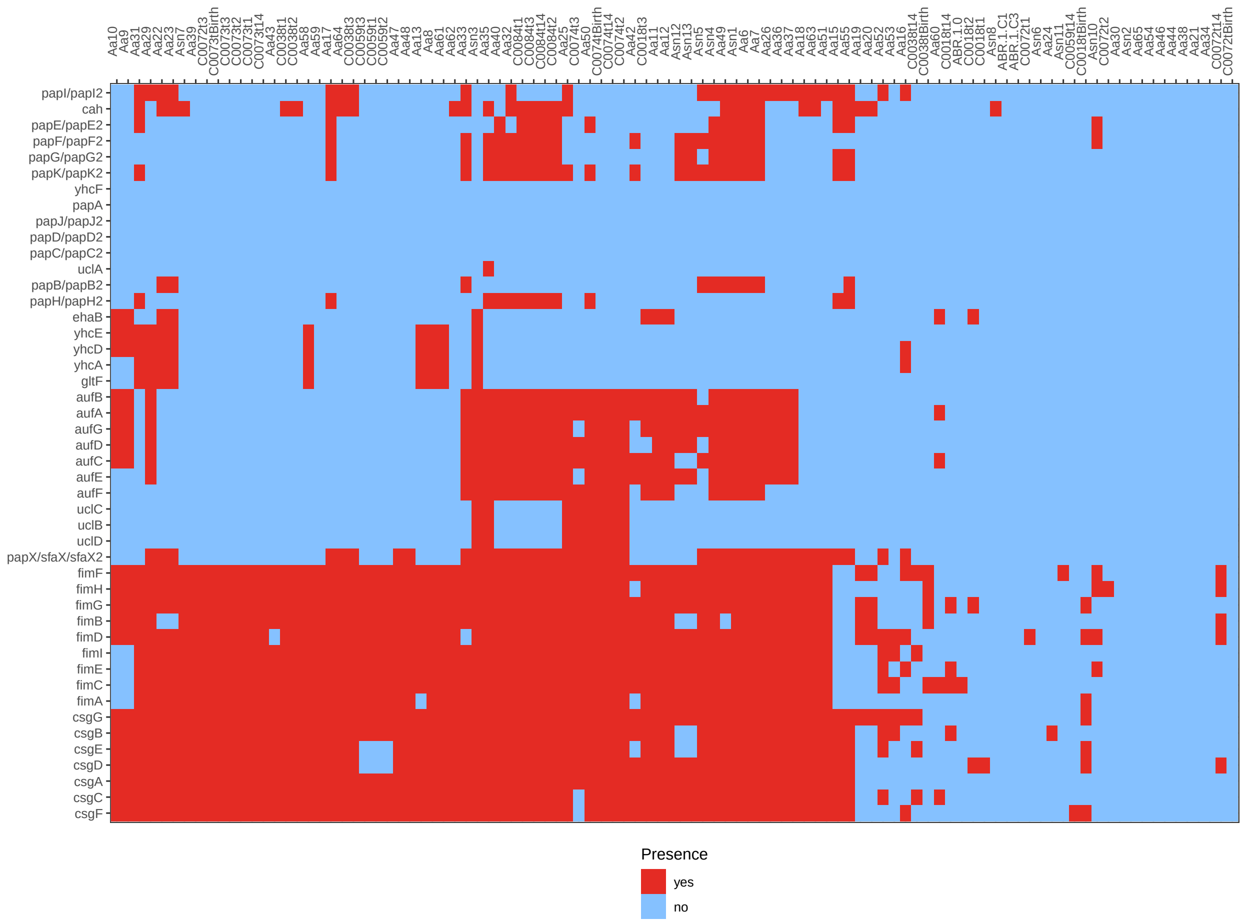

Analysis on system level

Get system information from gene presence. A system is considered as present if all of its genes are found.

```
system_df <- get_summary_table(presence_df)
```

Plotting results on system level

Again, you can plot all results:

```
get_summary_plot(presence_df[1:100, ])
```

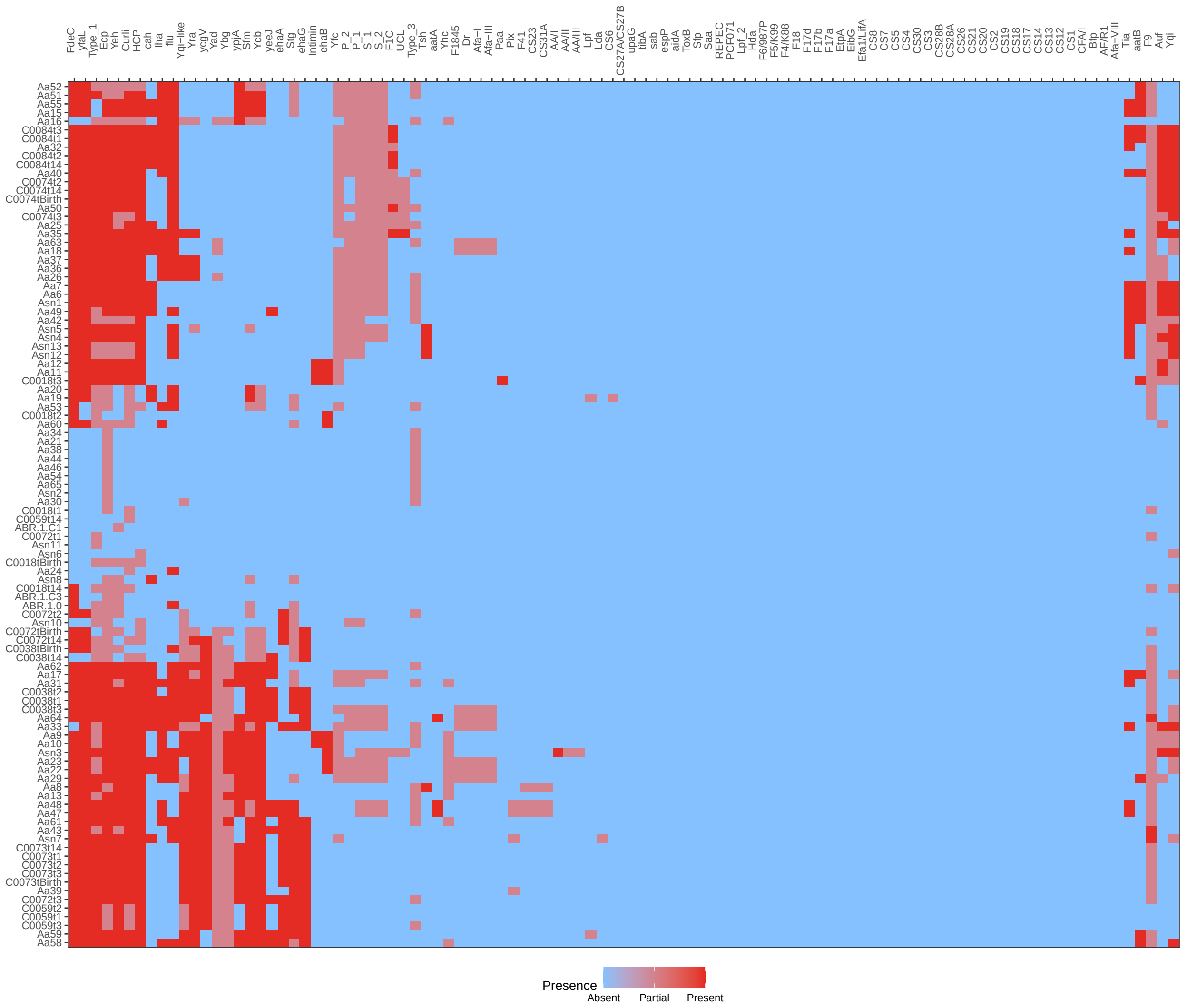

You can also skip systems that were not present in any of the analysed genomes using `hide_absent` argument:

```
get_summary_plot(presence_df[1:100, ],
                 hide_absent = TRUE)
```

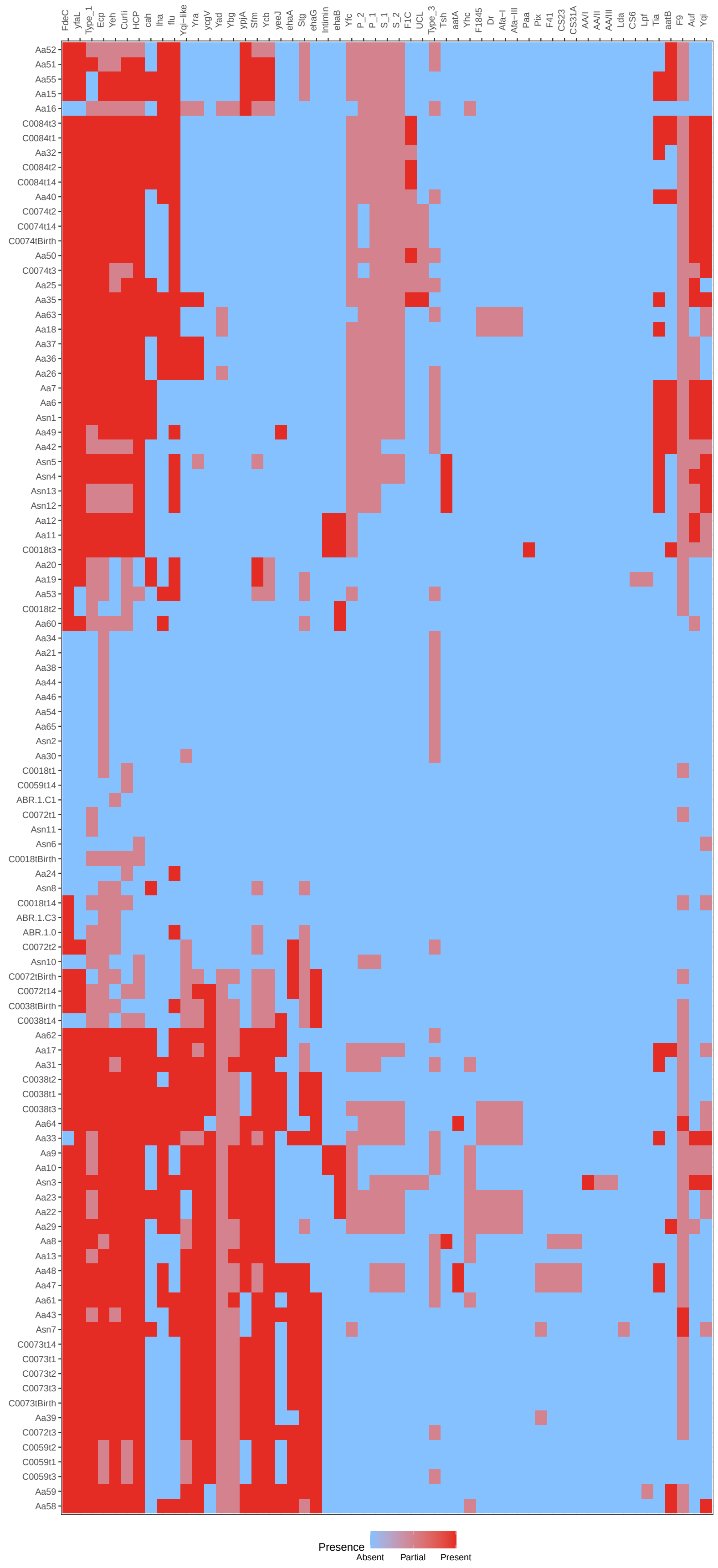
FdeC
yfaL
Type_1
Ecp
Yeh
Curli
HCP
cah
lha
flu
Yqi−like
Yra
ycgV
Yad
Ybg
ypjA
Sfm
Ycb
yeeJ
ehaA
Stg
ehaG
Intimin
ehaB
Yfc
P_2
P_1
S_1
S_2
F1C
UCL
Type_3
Tsh
aatA
Yhc
F1845
Dr
Afa−I
Afa−III
Paa
Pix
F41
CS23
CS31A
AA/I
AA/II
AA/III
Lda
CS6
Lpf
Tia
aatB
F9
Auf
Yqi
Aa52
Aa51
Aa55
Aa15
Aa16
C0084t3
C0084t1
Aa32
C0084t2
C0084t14
Aa40
C0074t2
C0074t14
C0074tBirth
Aa50
C0074t3
Aa25
Aa35
Aa63
Aa18
Aa37
Aa36
Aa26
Aa7
Aa6
Asn1
Aa49
Aa42
Asn5
Asn4
Asn13
Asn12
Aa12
Aa11
C0018t3
Aa20
Aa19
Aa53
C0018t2
Aa60
Aa34
Aa21
Aa38
Aa44
Aa46
Aa54
Aa65
Asn2
Aa30
C0018t1
C0059t14
ABR.1.C1
C0072t1
Asn11
Asn6
C0018tBirth
Aa24
Asn8
C0018t14
ABR.1.C3
ABR.1.0
C0072t2
Asn10
C0072tBirth
C0072t14
C0038tBirth
C0038t14
Aa62
Aa17
Aa31
C0038t2
C0038t1
C0038t3
Aa64
Aa33
Aa9
Aa10
Asn3
Aa23
Aa22
Aa29
Aa8
Aa13
Aa48
Aa47
Aa61
Aa43
Asn7
C0073t14
C0073t1
C0073t2
C0073t3
C0073tBirth
Aa39
C0072t3
C0059t2
C0059t1
C0059t3
Aa59
Aa58
Presence
Absent
Partial
Present

You can also modify plot colors by modifying `presence_col` and `absence_col` arguments:

```
get_summary_plot(presence_df[1:100, ],
                 hide_absent = TRUE,
                 presence_col = "#139e3d",
                 absence_col = "#bad1c1")
```

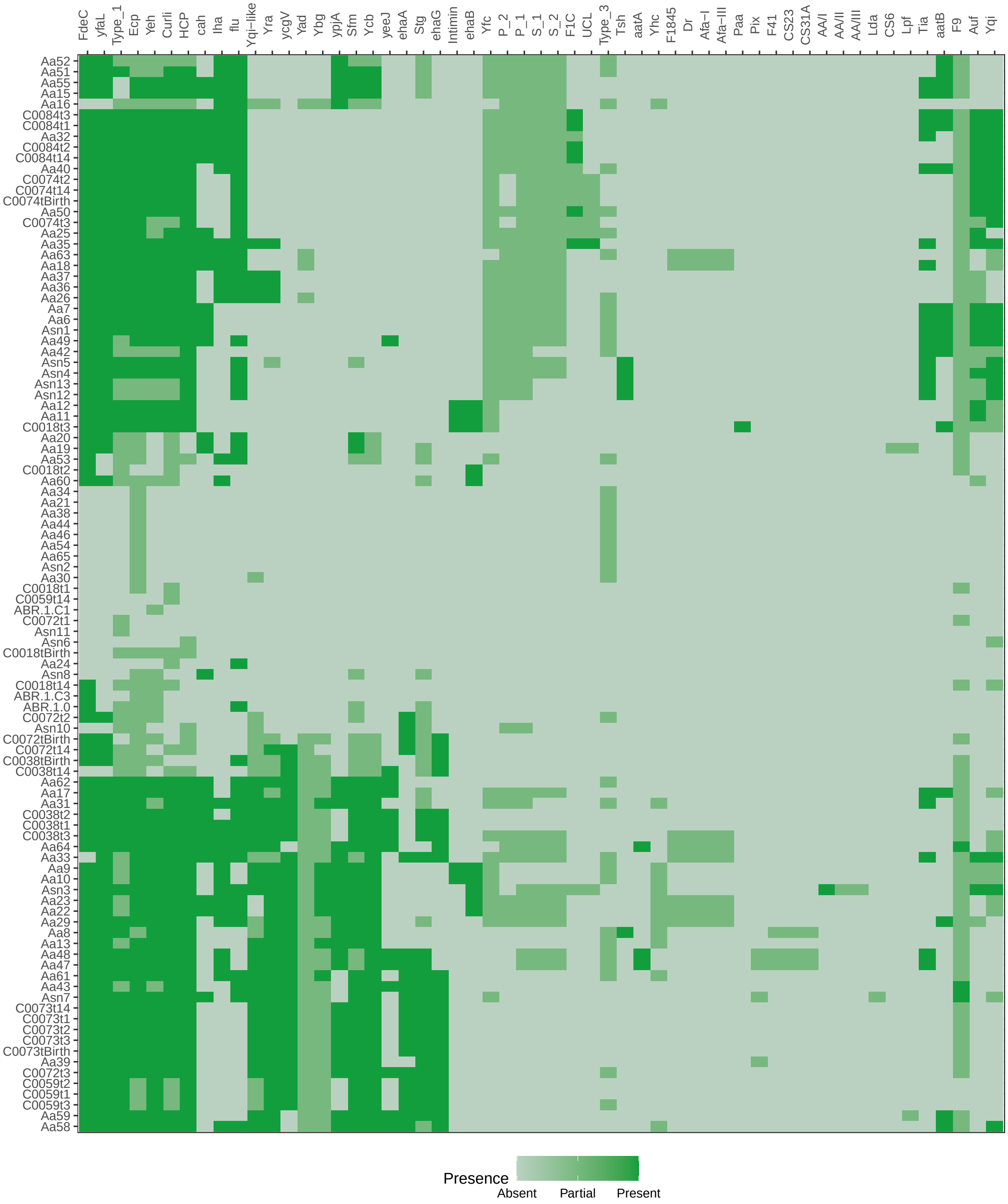

Run adhesiomeR analysis - relaxed version

The relaxed version of the search is meant for more broad searches and identification of potentially novel adhesins. In this setting, you can set identity percent and coverage thresholds.

We will use files published in Hildebrand et al. 2021.

Analysis on gene level

First, you need to run blast search on the gene catalogue. You can specify number of threads to use with `n_threads` argument:

```
library(adhesiomeR)
blast_results <- get_blast_res("compl.incompl.95.fna1", n_threads = 12)
```

Save blast results to csv file for further processing

```
write.csv(blast_results, "gc_blast_results.csv", row.names = FALSE)
```

Extract subset of data from abundance matrix. Notice that this step does not use R but bash:

```
# Extract gene names with hits to adhesins
cut -d$'\t' -f 1 gc_blast_results.csv | uniq | grep -v "Query" > genes.txt

# Extract gene abundance in samples
zcat Matrix.mat.gz | head -n 1 > gene_abundance.txt
zcat Matrix.mat.gz | sed -nf <(sed 's/$/p/' genes.txt) >> gene_abundance.txt
```

You can also use these commands from within R using system function.

```
# Extract gene names with hits to adhesins
system("cut -d$'\t' -f 1 gc_blast_results.csv | uniq | grep -v 'Query' > genes.txt")

# Extract gene abundance in samples
system("zcat Matrix.mat.gz | head -n 1 > gene_abundance.txt")
system("zcat Matrix.mat.gz | sed -nf <(sed 's/$/p/' genes.txt) >> gene_abundance.txt")
```

The next step is to trace back the results obtained for the gene catalogue to individual samples based on abundance matrix:

```
# Define path to the subset of abundance matrix
ab_mat <- "gene_abundance.txt"

# Extend blast results
extended_blast_res <- gc_to_sample(blast_results, ab_mat)
```

The next step is to get gene presence information from extended blast results. Here, you can set the thresholds for considering gene as present or absent. By default, adhesiomeR uses 75% thresholds for both. In the resulting table, 1 indicates gene presence and 0 its absence.

```
presence_rel <- get_presence_table_relaxed(blast_res = extended_blast_res,
                                           n_threads = 8)
```

You can modify the default thresholds using `identity` and `coverage` arguments:

```
presence_rel2 <- get_presence_table_relaxed(blast_res = extended_blast_res,
                                            identity = 90,
                                            coverage = 90,
                                            n_threads = 8)
```

If you wish to see only genes that were found in at least one file, you can set `add_missing` argument to `FALSE`. Note that by default the results include all genes from the adhesiomeR database.

```
presence_rel3 <- get_presence_table_relaxed(blast_res = extended_blast_res,
                                            n_threads = 8,
                                            add_missing = FALSE)
```

Plotting results on gene level

You can easily plot the presence/absence of adhesin genes. For simplicity (and due to the size of the full plot), we will plot only a few systems: type 1, Auf, Yhc, Pix, UCL fimbriae, ehaB, cah and paa. By default (without specifying `systems` argument), genes from all systems will be plotted. Note that if you analyse more than one genome, the results on a heatmap are clustered for more clear visualisation. Due to the size of the gene catalogue, we will use only first 100 rows of data for this plot.

```
get_presence_plot(presence_table = presence_rel[1:100, ],
                  systems = c("Type_1", "Auf", "Yhc", "Curli",
                              "P_1", "UCL", "ehaB", "cah", "paa"))
```

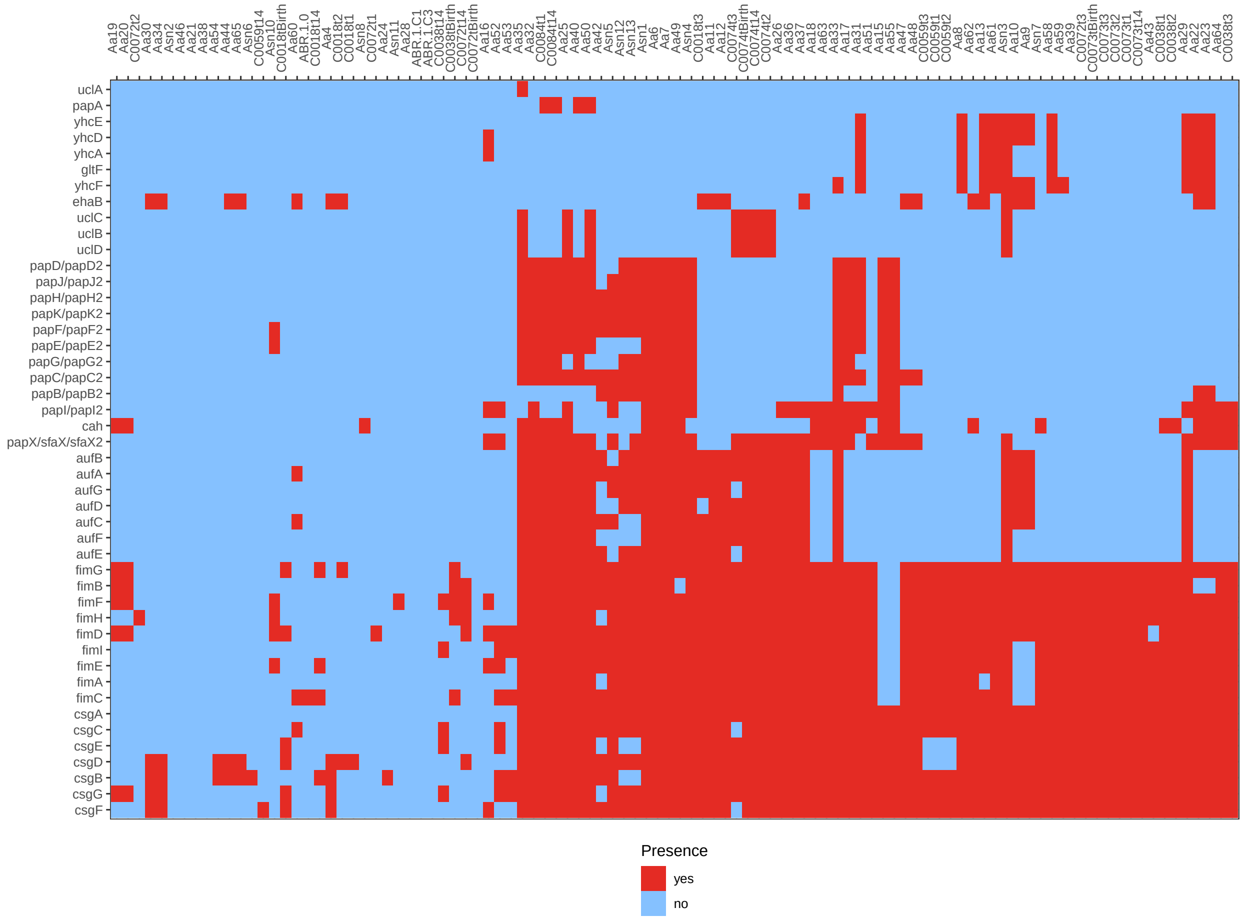

The plot above used results obtained with default thresholds (75% identity and coverage). Let's see how it looks like when we plot results obtained with thresholds set to 90%.

```
get_presence_plot(presence_table = presence_rel2[1:100, ],
                  systems = c("Type_1", "Auf", "Yhc", "Curli",
                              "P_1", "UCL", "ehaB", "cah", "paa"))
```

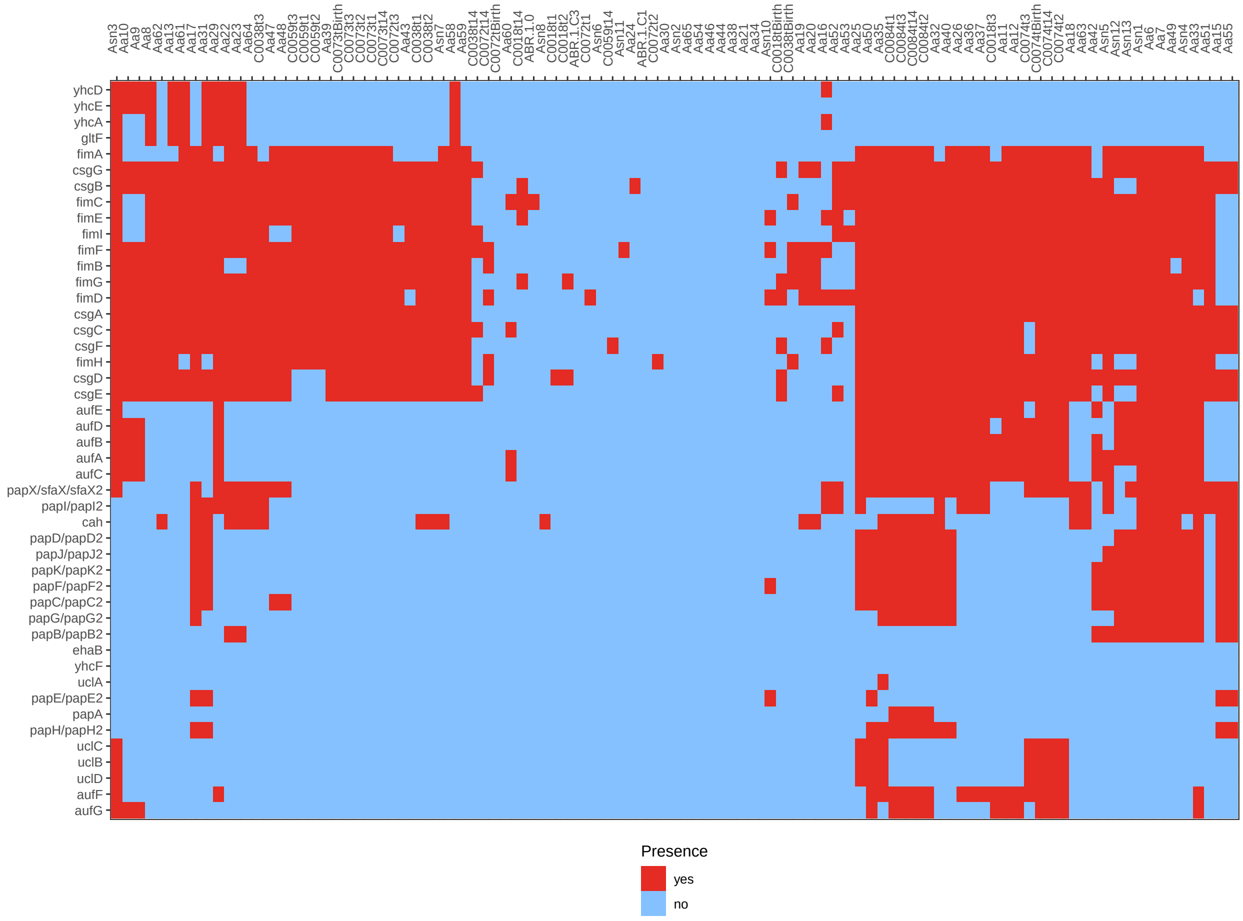

Analysis on system level

Get system information from gene presence. A system is considered as present if all of its genes are found.

```
system_rel <- get_summary_table(presence_rel)
```

Plotting results on system level

Again, you can plot all results:

```
get_summary_plot(presence_rel[1:100, ])
```

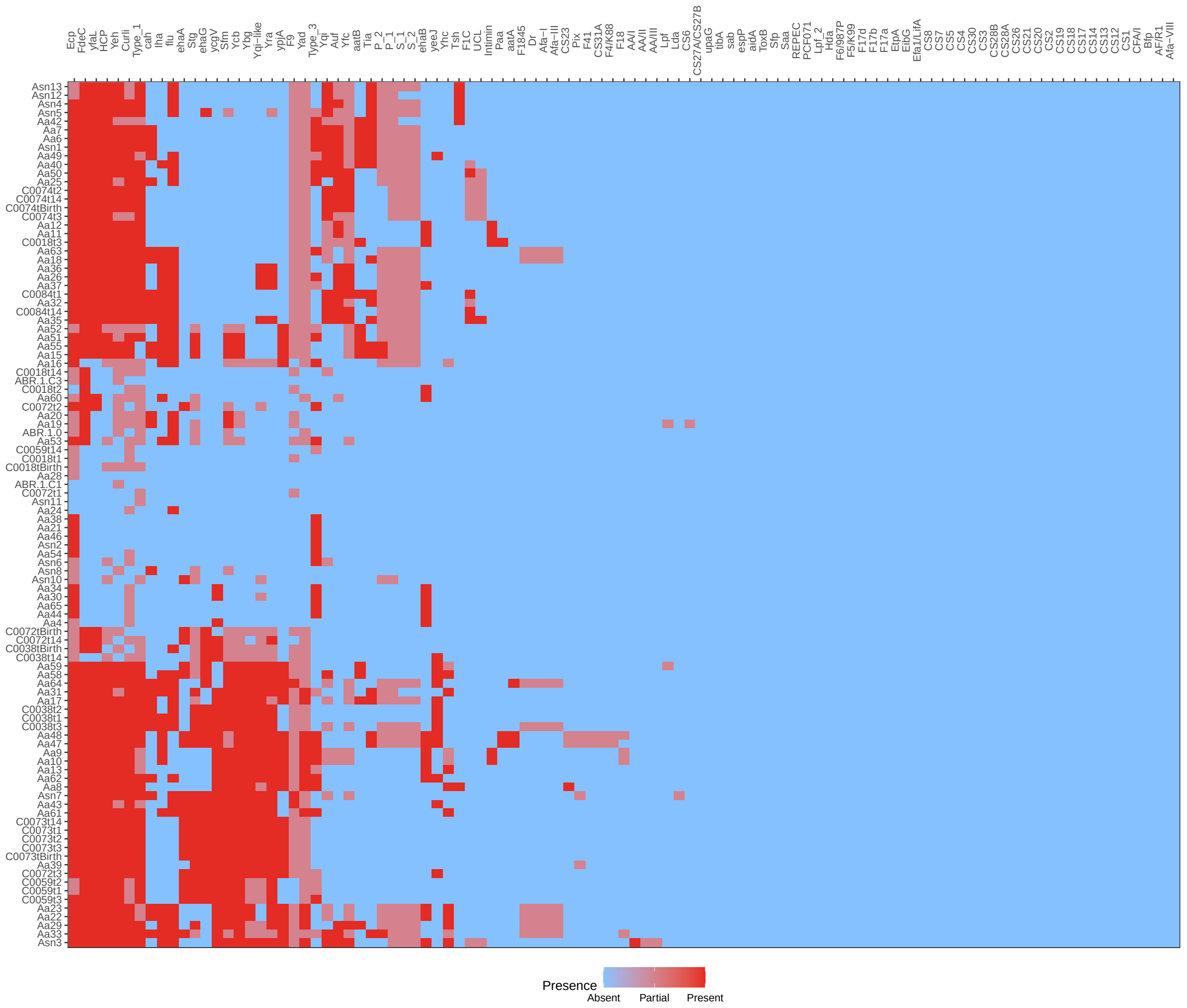

You can also skip systems that were not present in any of the analysed genomes using `hide_absent` argument:

```r
get_summary_plot(presence_rel[1:100, ],
                 hide_absent = TRUE)
```

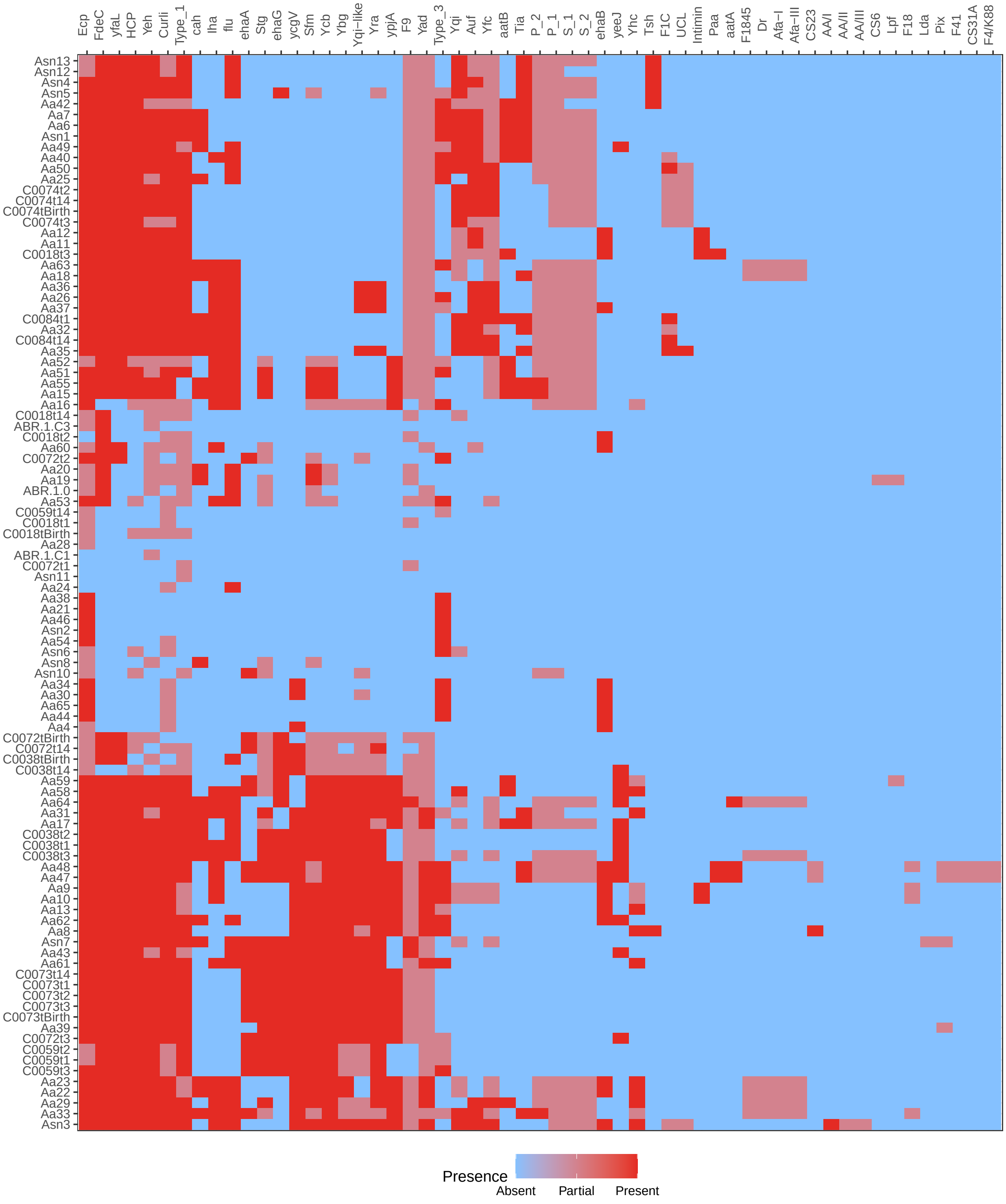

You can also modify plot colors by modifying `presence_col` and `absence_col` arguments:

```
get_summary_plot(presence_rel[1:100, ],
                 hide_absent = TRUE,
                 presence_col = "#139e3d",
                 absence_col = "#bad1c1")
```

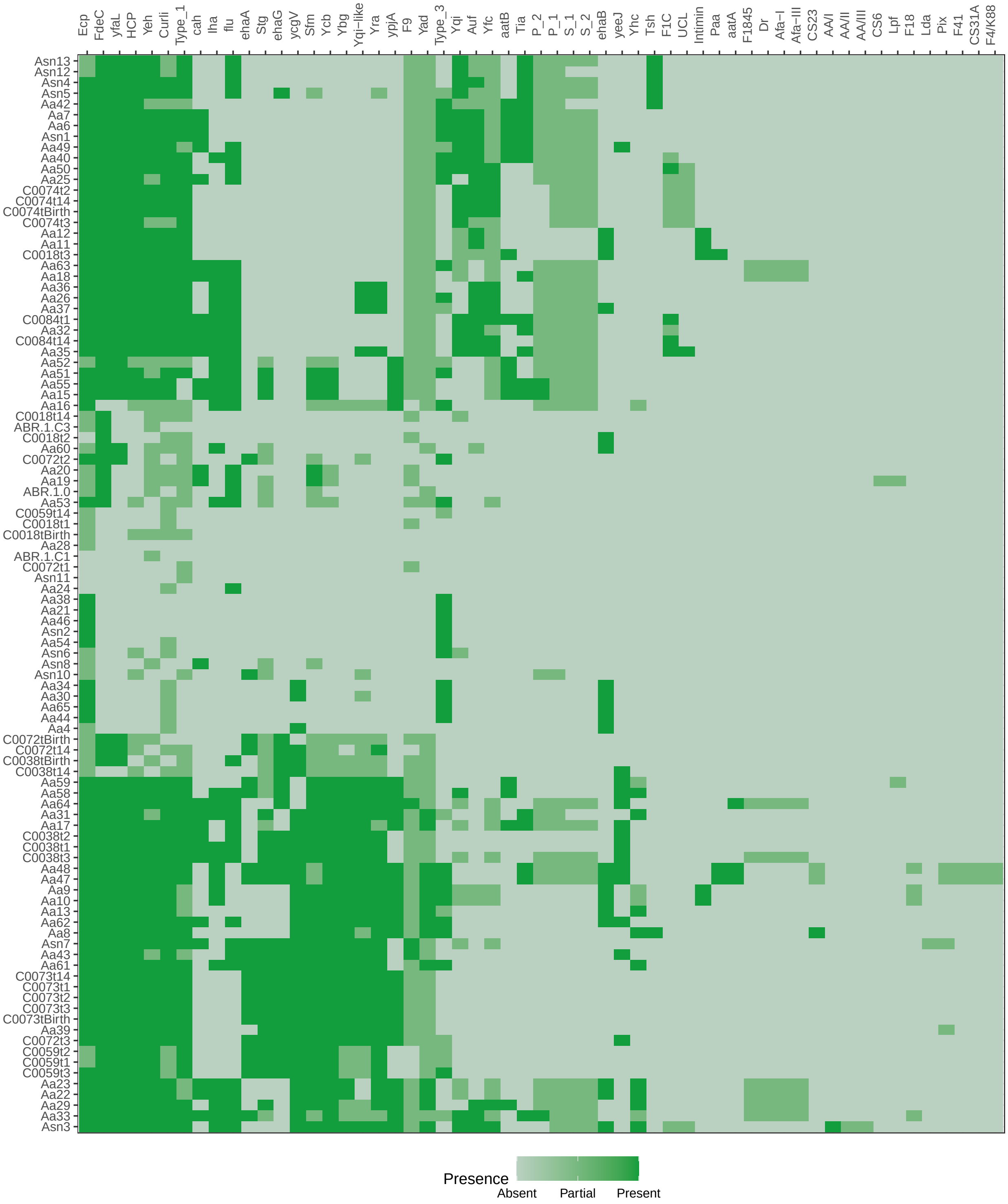

Please note that due to the nature of the gene catalogue, it is not possible to determine adhesin gene copy number using this approach. It is also not recommended to use profile and cluster assignment as they have been developed to be used mainly with individual genome assemblies.
